# Supplementary material for: The unfolded protein response regulates ER exit sites via SNRPB-dependent RNA splicing and contributes to bone development
Source: EMBO J. 2024 Aug 19;43(19):4228–47. doi: 10.1038/s44318-024-00208-z (PMC11445528; doi:10.1038/s44318-024-00208-z)
Supplement: Supplementary file 1 — Appendix [file 44318_2024_208_MOESM1_ESM.pdf]

Appendix for

The unfolded protein response regulates ER exit sites via SNRPB-dependent RNA splicing and contributes to bone development.

Appendix figures ToC

Appendix Figure S1 ……………………………………………..1
Appendix Figure S2 ……………………………………………..2
Appendix Figure S3 ……………………………………………..3
Appendix Figure S4 ……………………………………………..4
Appendix Figure S5 ……………………………………………..5
Appendix Figure S6 ……………………………………………..6
Appendix Figure S7 ……………………………………………..7
Appendix Figure S8 ……………………………………………..8
Appendix Figure S9 ……………………………………………..9
Appendix Figure S10 ……………………………………………..10
Appendix Figure S11 ……………………………………………..11

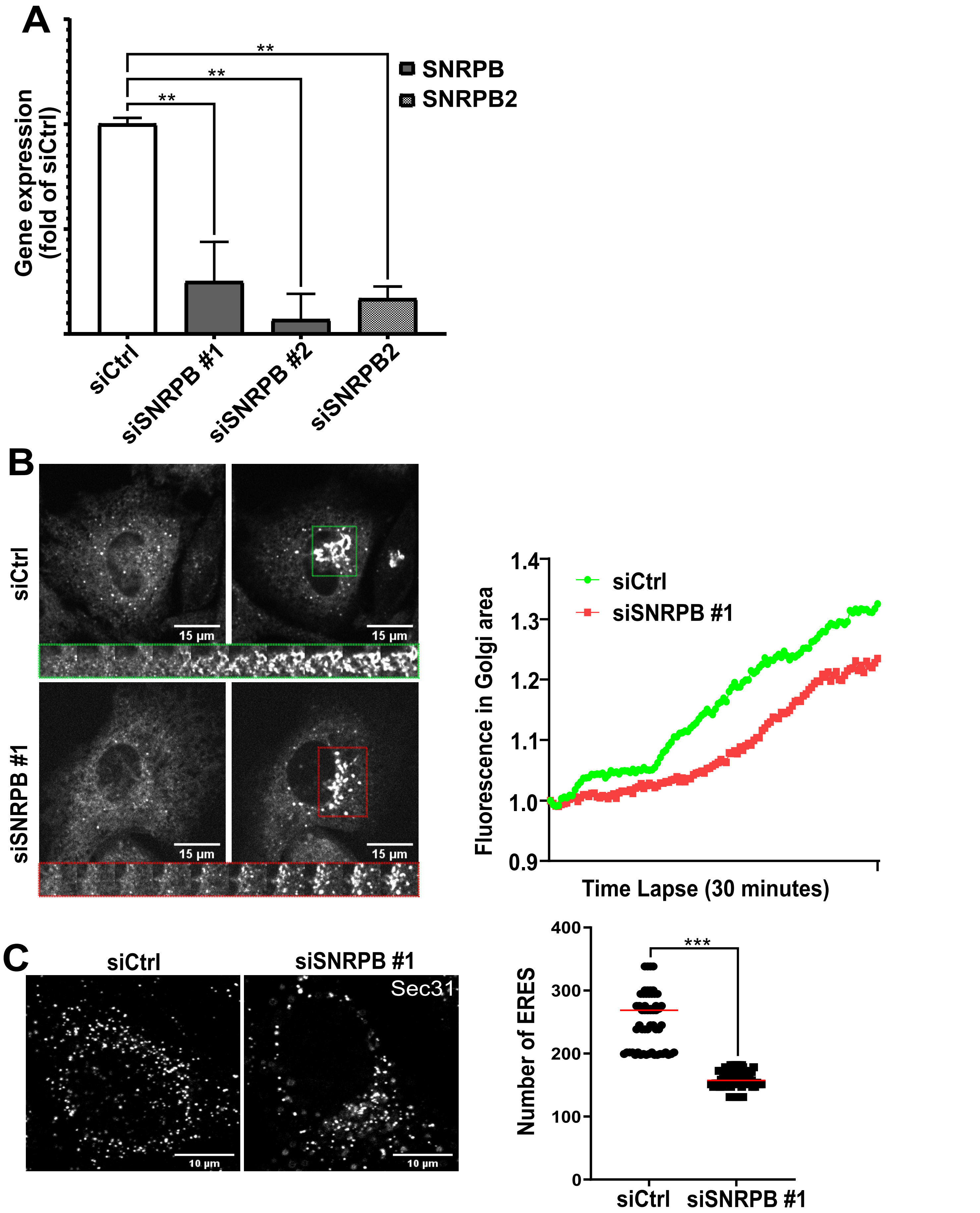

Appendix Figure S1. ***A***, HeLa cells were lysed 72 h after siRNA transfection with the indicated siRNA followed by qRT-PCR showing that the knockdown of SNRPB and SNRPB2 works. ***B,*** HeLa cells stably expressing mCherry tagged mannosidase-II RUSH construct were transfected with siRNA against SNRPB (siSNRPB) or with a non-targeting control siRNA (siCtrl). After 72 h, cells were treated with biotin (40 µM) for the indicated and the cells were imaged for 30 min at 37C and in the presence of 5% CO_2. ***C***, Effect of SNRPB knockdown on ERES number. Experiment performed as in Figure 2A. Data are from 3 independent experiments. *** indicates p<0.001, unpaired two-tailed t-test.

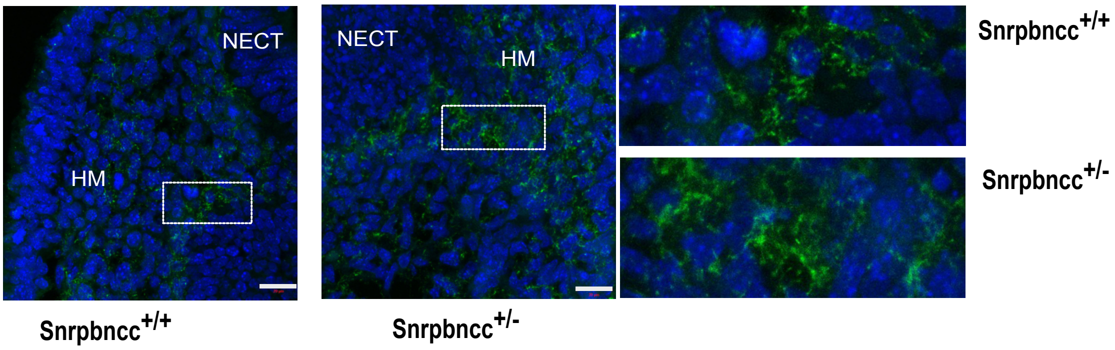

Appendix Figure S2. Immunofluorescence staining of collagen (green) and nuclei (blue) in the head region of E9.5 embryos of *Snrpb* control (*Snrpb*$^{+/+}$*; Wnt*$^{tg/+}$) and mutant (*Snrpb*$^{ncc/+}$) embryos. Scale bar = 20 µm

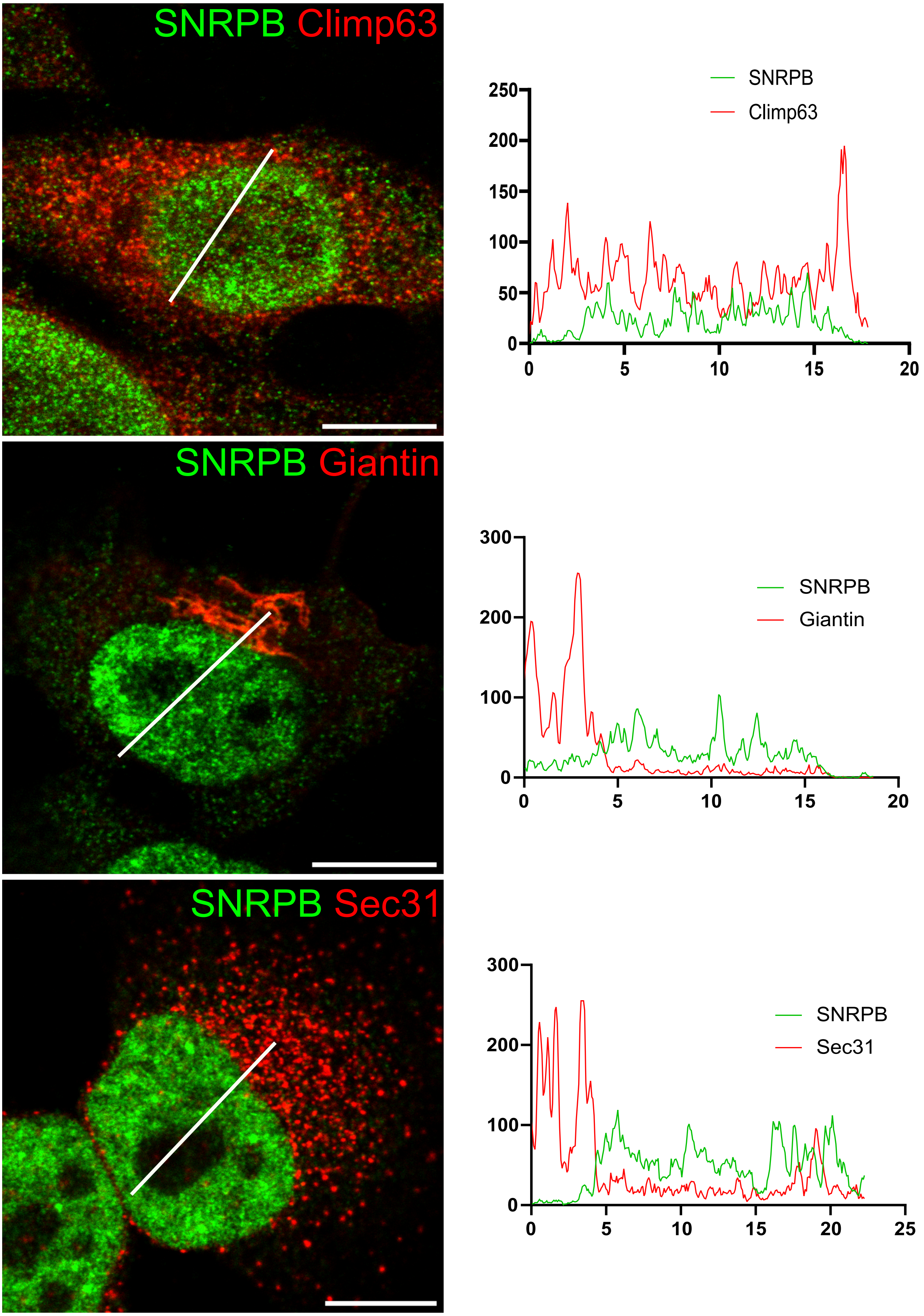

Appendix Figure S3. Localization of SNRPB. HeLa cells were fixed and stained against SNRPB in combination with an ER marker CLIMP63, the Golgi marker giantin and the ERES marker Sec31. The line profile is displayed to the right side of the images. Scale bar = 10 µm.

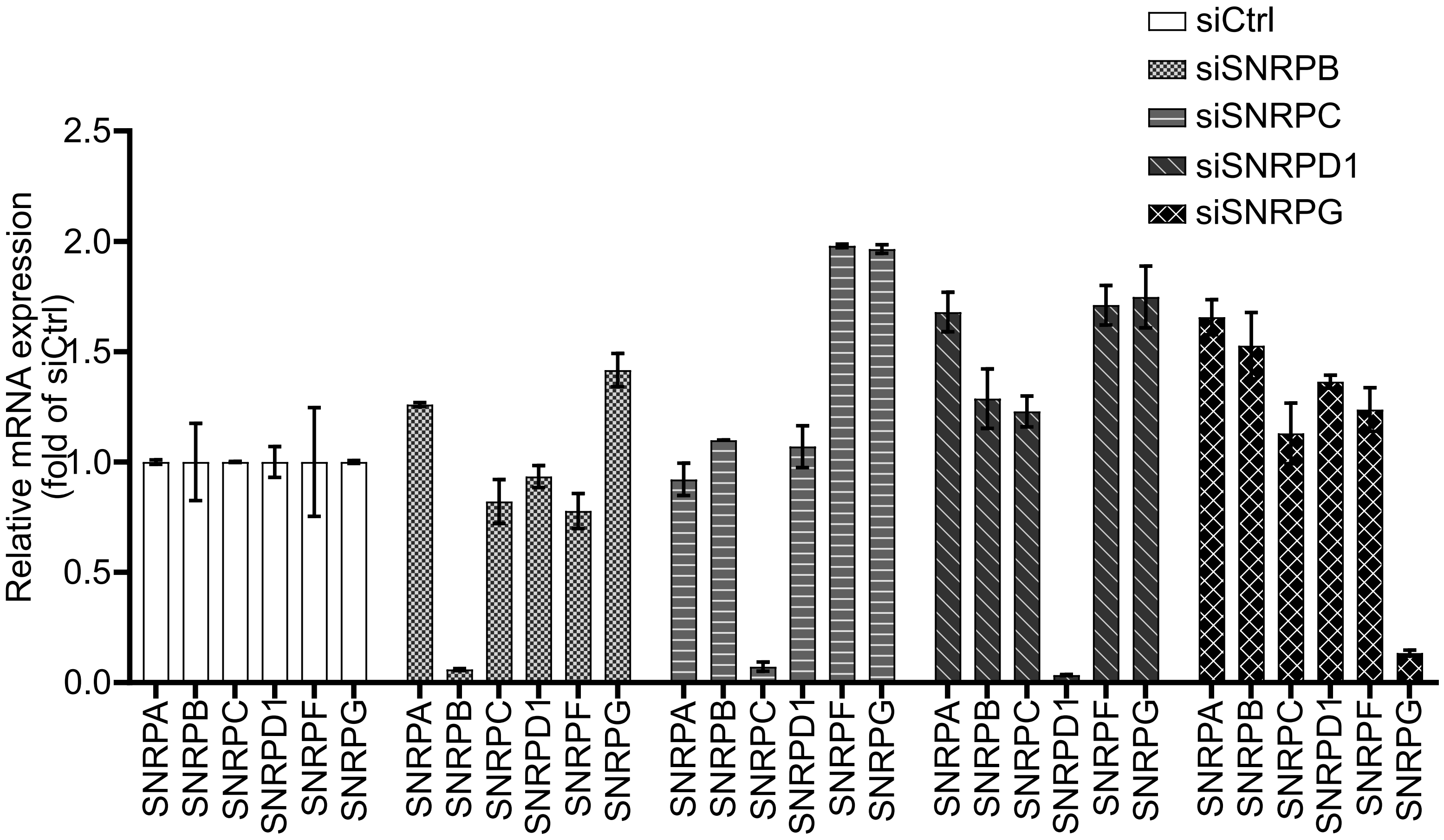

Appendix Figure S4. HeLa cells were transfected with siRNA against the indicated transcripts. siCtrl indicates transfection with non-targeting control siRNA. After 72 h, qPCR was performed to measure the levels of the mRNAs of the Sm-ring components SNRPB, SNRPC, SNRPD1, SNRPF and SNRPG as well as for the non-Sm-ring SNRPA. Data are ± SD from 3 experiments.

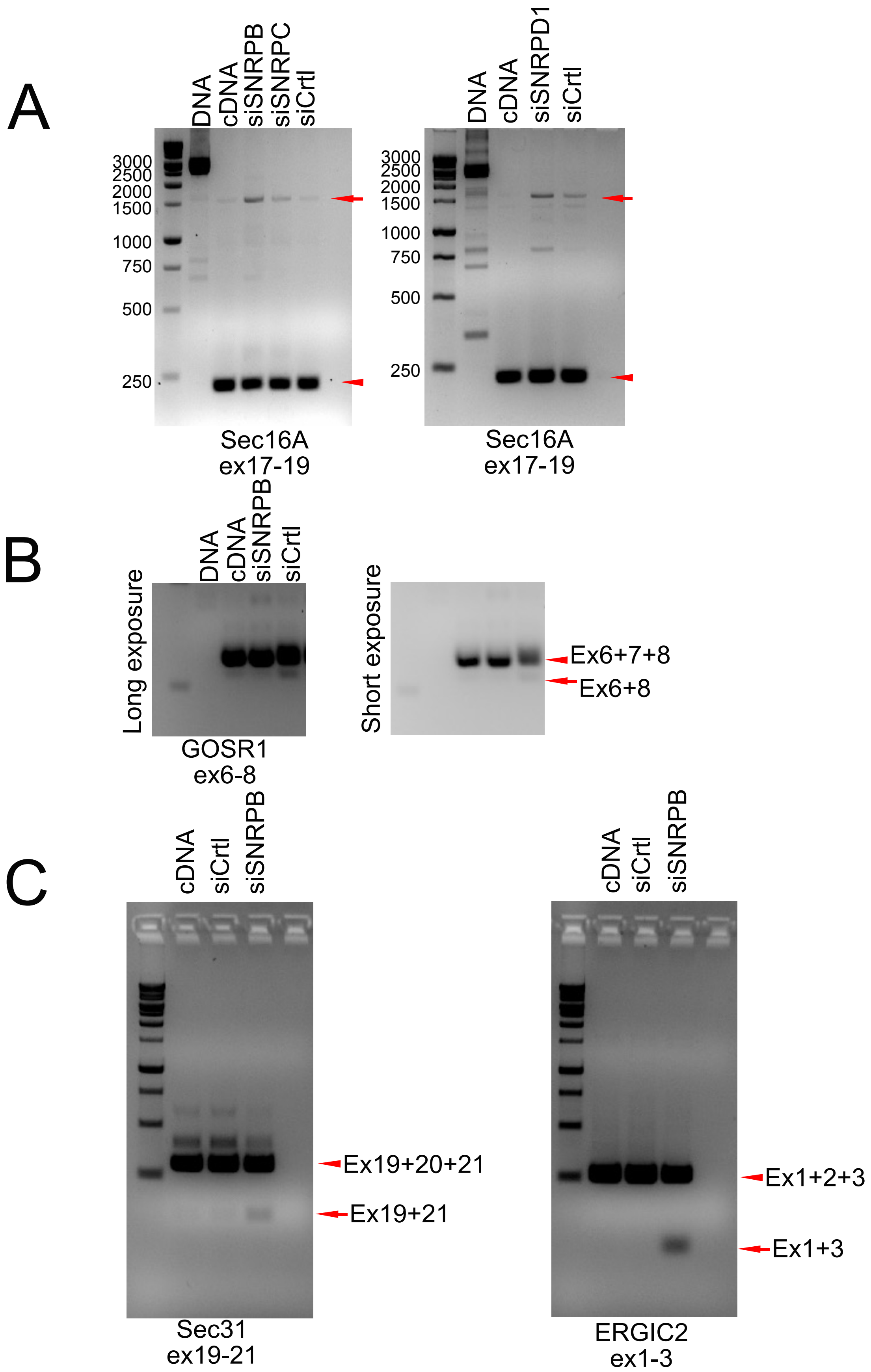

Appendix Figure S5. ***A,*** HeLa cells were transfected with non-targeting siRNA (siCtrl), or siRNA against SNRPB, SNRPC, or SNRPD1. After 72 h, cells were lysed and PCR was performed for a region spanning exons 17-19 of Sec16A. The unspliced product is indicated with an arrow and the spliced product with an arrow head. Furthermore, PCR was performed for a region spanning exons 6-8 of Sec16A. The unspliced product (arrow) is predicted to be at 952 bp and the completely spliced product is 331 bp (arrow head). ***B,*** HeLa cells were transfected with non-targeting siRNA (siCtrl), or siRNA against SNRPB. After 72 h, cells were lysed and PCR was performed for a region spanning exons 6-9 of GOSR1. Two exposures of the gel are shown. The arrow head indicated the position of a PCR product with exons 6 to 8 and the arrow indicates a PCR product missing exon 7, which was affected in our RNAseq analysis. ***C,*** HeLa cells were transfected with non-targeting siRNA (siCtrl), or siRNA against SNRPB. After 72 h, cells were lysed and PCR was performed for a region spanning exons 19-21 of Sec31 as well as exons 1-2 of ERGIC2. Arrows and arrowheads indicate the positions of the amplicons with or without exon skipping.

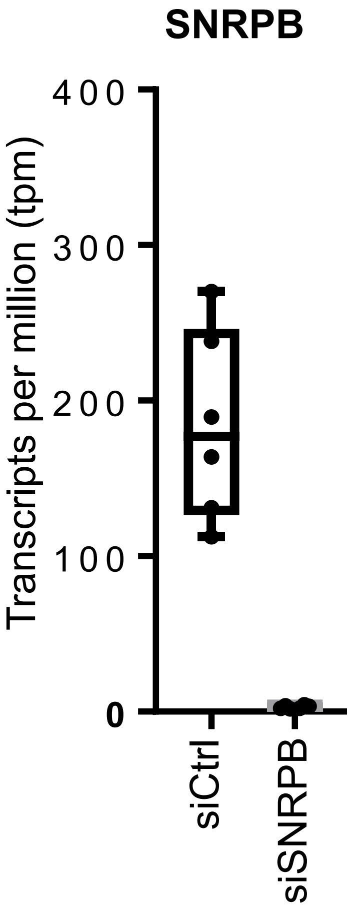

Appendix Figure S6. Graph showing the levels of SNRPB in HeLa cells transfected with siRNA against SNRPB or a non-targeting control (siCtrl). Cells were lysed and used for RNAseq experiments. The data are from 3 experiments performed in duplicates. Data were represented as box plots. The interquartile range is presented by a rectangular box with the median as the center of the box. Whiskers indicate the min. to max. range.

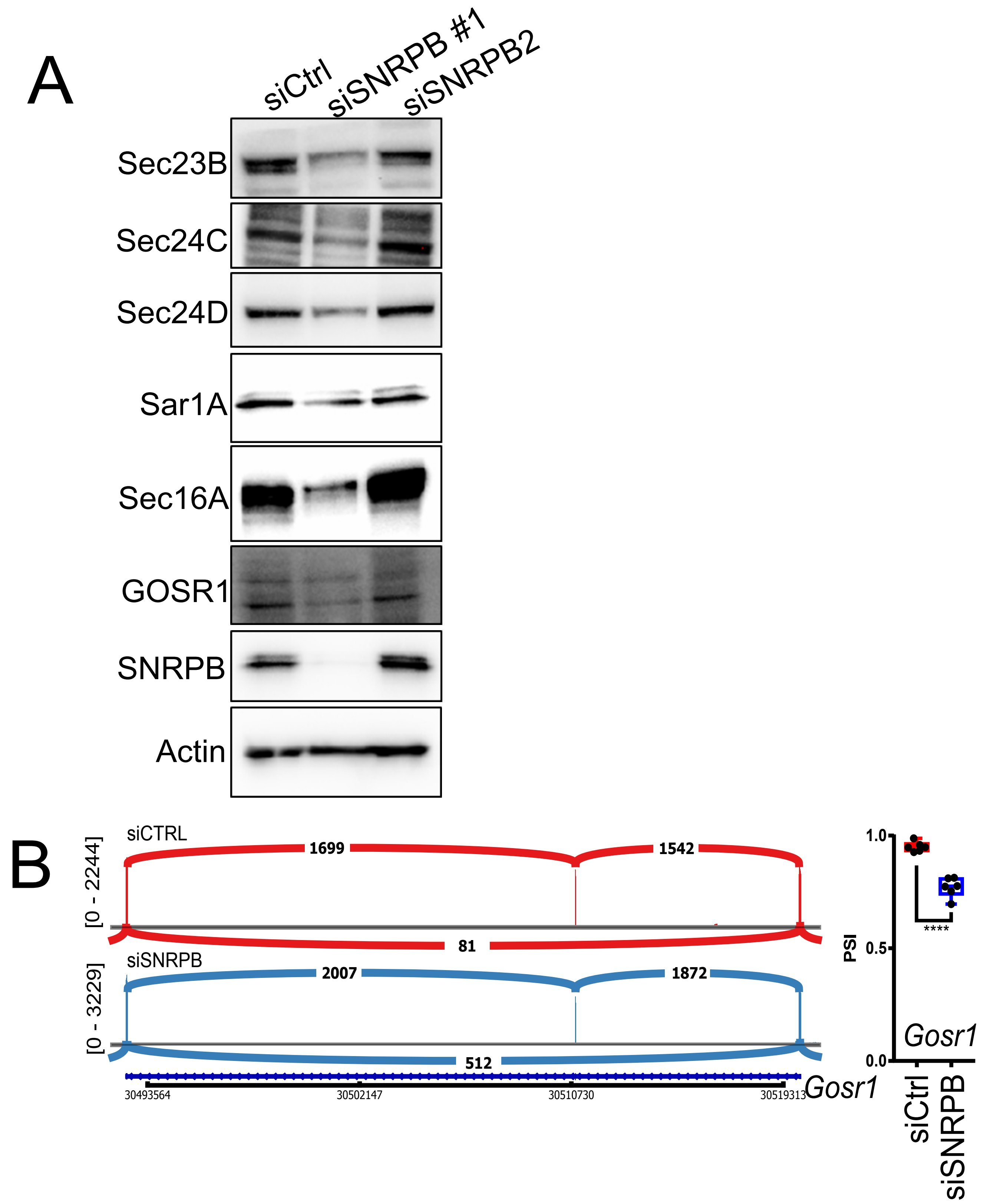

Appendix Figure S7. SNRPB depletion regulates protein levels of ERES components. ***A,*** HeLa cells were transfected with siRNAs against SNRPB or SNRPB2 or with a non-targeting control siRNA (siCtrl). After 72 h, cells were lysed and immunoblotted as indicated. ***B,*** Sashimi blots highlighting SNRPB depletion induced changes in exon inclusion. Summarized are all reads from siCTRL (red) or siSNRPB (blue). Pilled sequencing coverage is indicated on the y-axis (number of reads per position). Junction reads are shown as lines (minimum junction reads per junction shown is set to 10). Below the genomic region as well as the exon intron structure surrounding the effected exons is shown. On the right, Whippet-derived percentage spliced in values (PSI) are shown (n=6). T-test derived significance is indicated by asterisks ****p<0.0001. Data were represented as box plot. The interquartile range is presented by a rectangular box with the median as the center of the box. Whiskers indicate the min. to max. range. Data points are displayed within the plot.

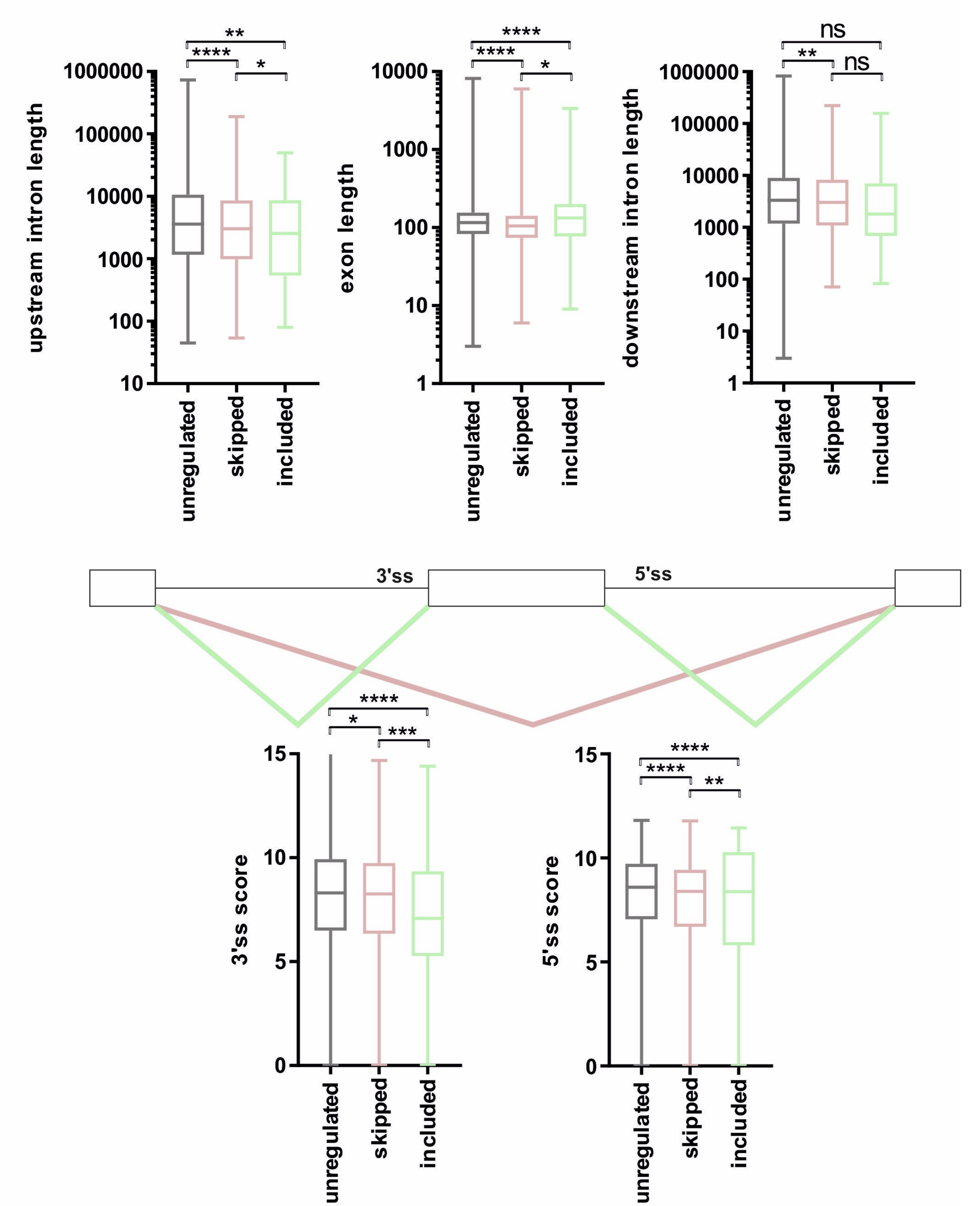

Appendix Figure S8. Features of SNRPB regulated exons. Unregulated (grey), SNRPB depletion skipped (red) and included (green) exons were compared for upstream intron length, exon length, downstream intron length, 3'splice-site and 5'-splice site strength. A scheme is shown in the center. Statistical significance was determined by unpaired t-test and is indicated by asterisks *<0.05, **<0.01, ***<0.001, ****<0.0001. The data are from 3 experiments performed in duplicates. Data were represented as box plots. The interquartile range is presented by a rectangular box with the median as the center of the box. Whiskers indicate the min. to max. range.

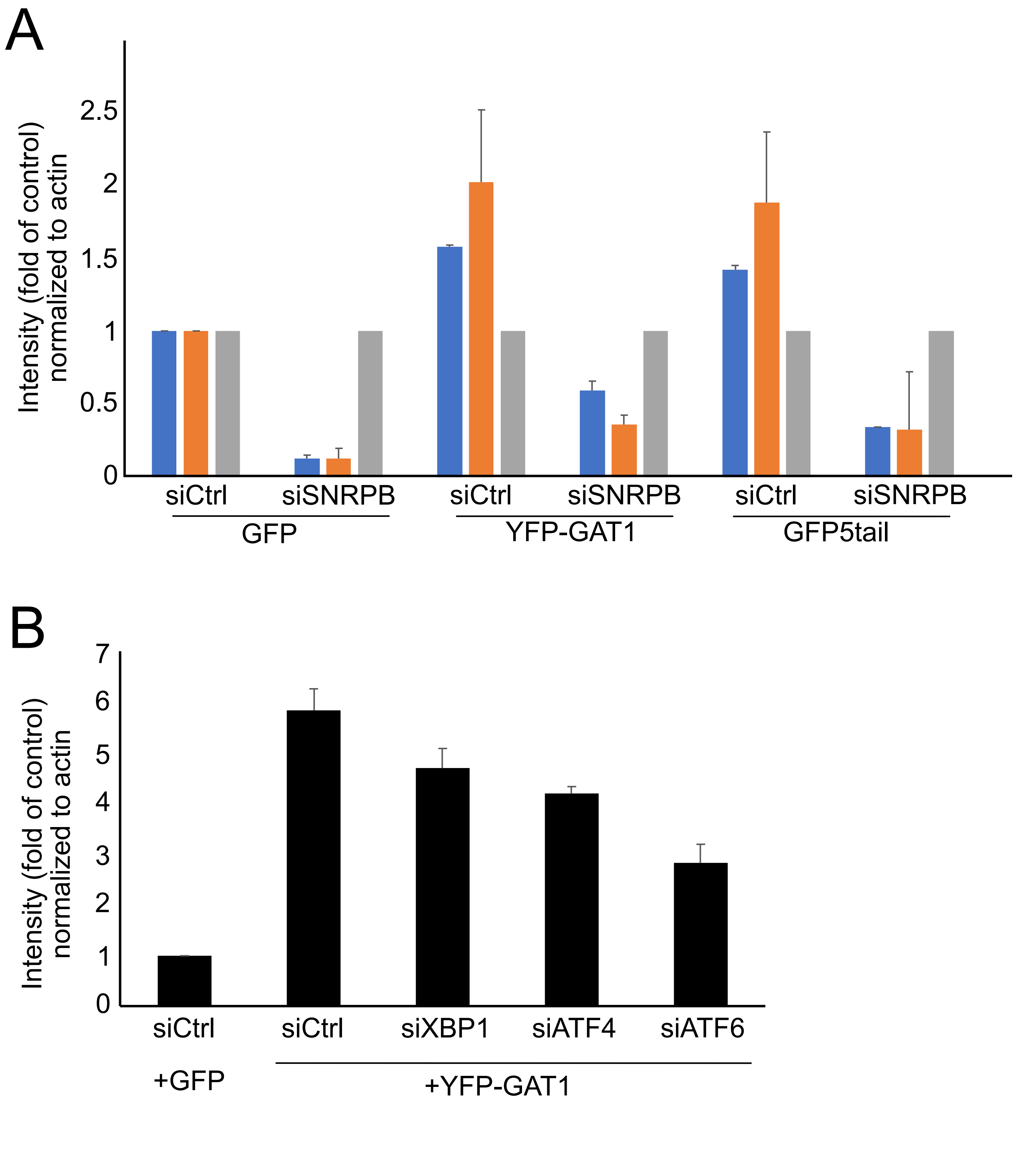

Appendix Figure S9. ***A,*** Densitometric quantification of the immunoblots from Figure 7A. ***B,*** Densitometric quantification of the immunoblots from Figure 7D. All data are ± SD from 3 independent experiments.

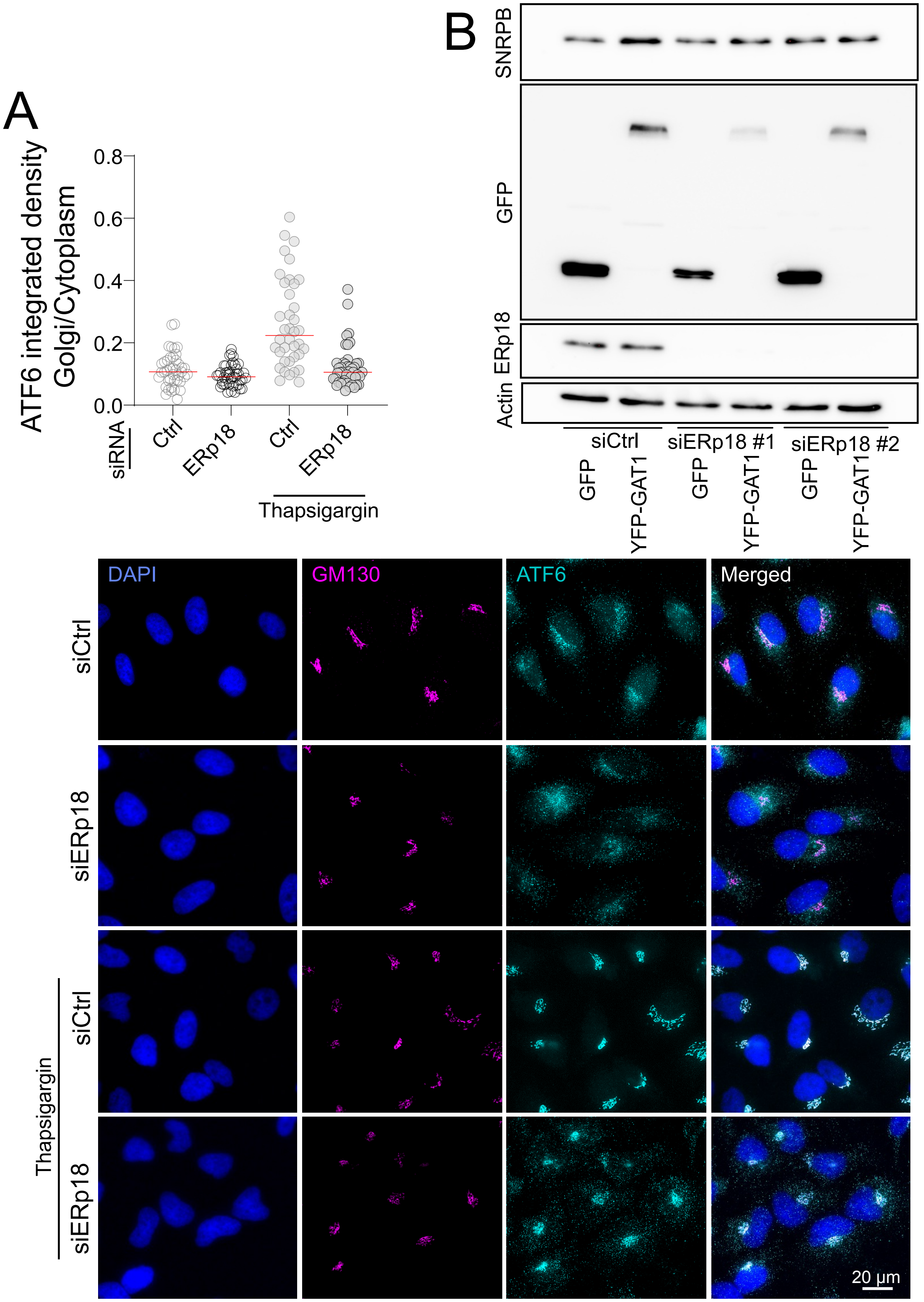

Appendix Figure S10. ERp18 regulates induction of SNRPB. ***A,*** HeLa cells were transfected with non-targeting siRNA (siCtrl) or with siRNA against ERp18. After 72 h, cells were treated either with DMSO or with 2M thapsigargin for 6 h. Subsequently, cells were fixed and stained with DAPI, anti-GM130 and anti-ATF6. The graph shows the quantification of the intensity of ATF6 signal in the Golgi region, in relation to the rest of the cell. ***B,*** HeLa cells were transfected with non-targeting siRNA (siCtrl) or with two different siRNAs against ERp18. After 48 h, cells were transfected with plasmids encoding GFP or YFP-tagged GAT1. After 24 h, cells were lysed and immunoblotted as indicated.

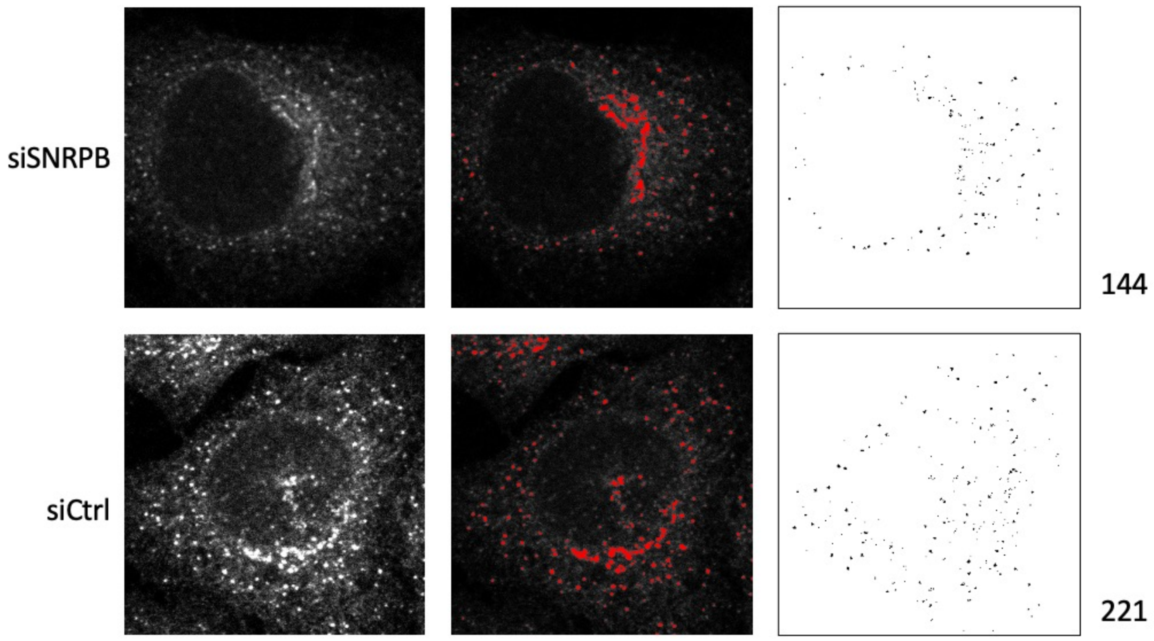

Appendix Figure S11. Screenshots of the analysis of the number of peripheral ERGIC puncta from the two cells from Figure 1C. The count shows that the control cell has 221 such structures while we counted 144 puncta in the SNRPB depleted cell.
