# Supplementary figures and images for: The unfolded protein response regulates ER exit sites via SNRPB-dependent RNA splicing and contributes to bone development
Source: EMBO J. 2024 Aug 19;43(19):4228–47. doi: 10.1038/s44318-024-00208-z (PMC11445528; doi:10.1038/s44318-024-00208-z)

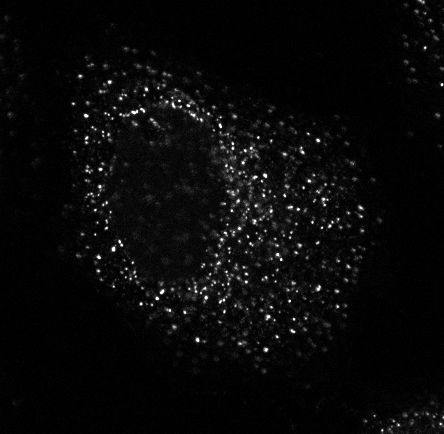

Supplement: Supplementary file 7 — Source data Fig. 2 [file 44318_2024_208_MOESM7_ESM.zip › Figure2/Figure 2A-Hela_siSNRPBsig_Sec13-Sec13_03.czi - C=1-1.czi.tif]

## Slide 1
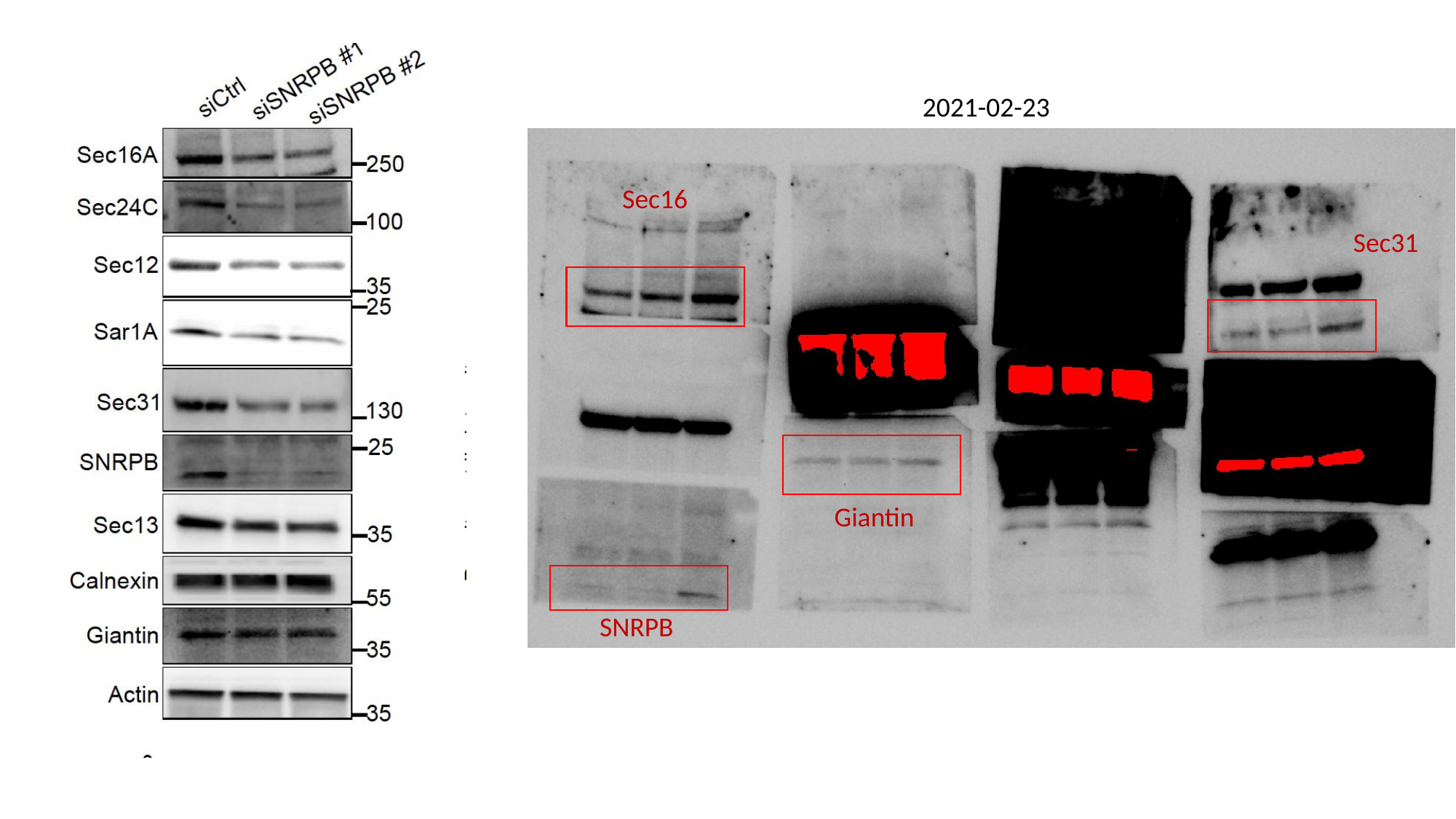

2021-02-23
Sec16
Sec31
Giantin
SNRPB

## Slide 2
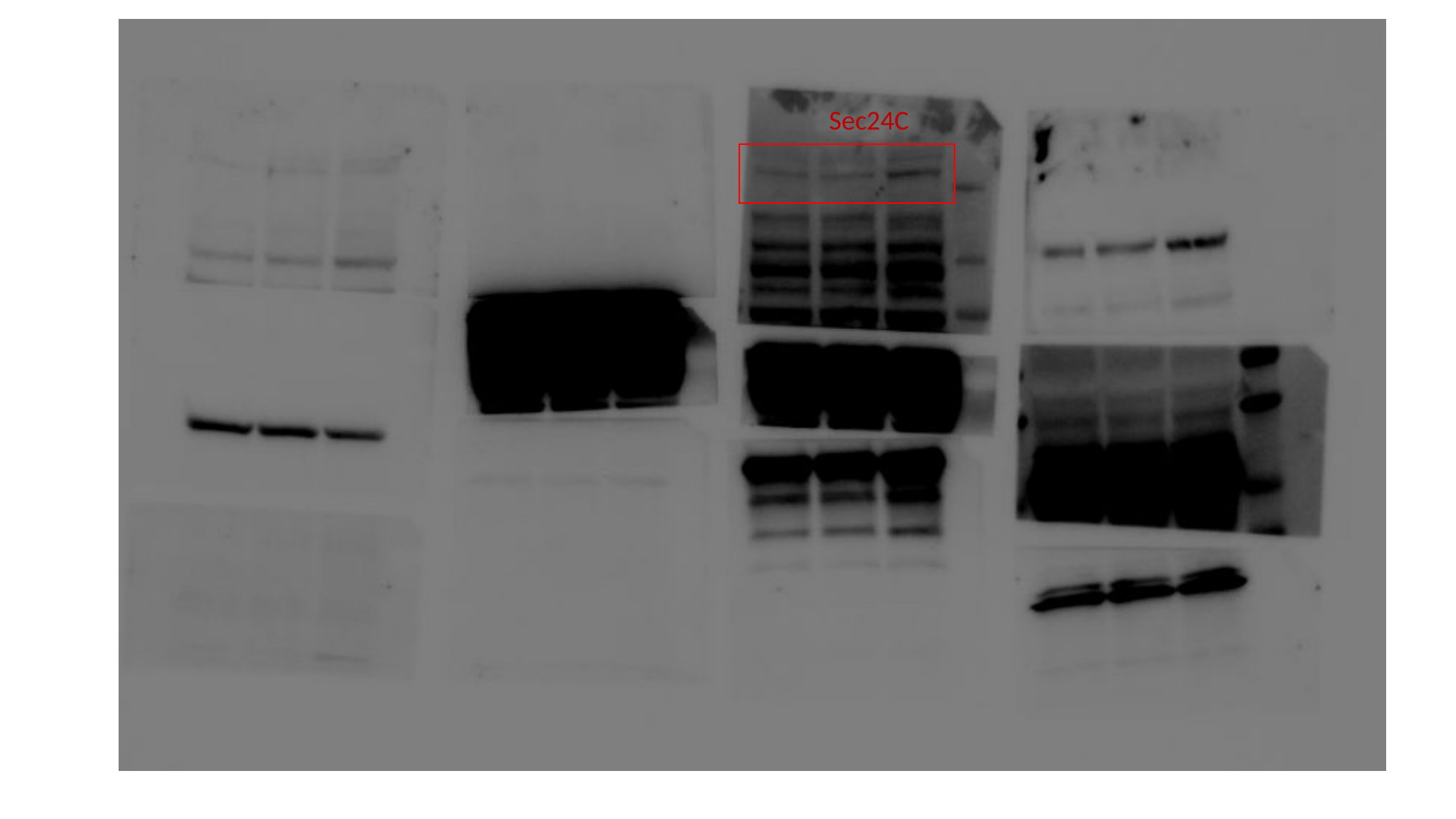

Sec24C

## Slide 3
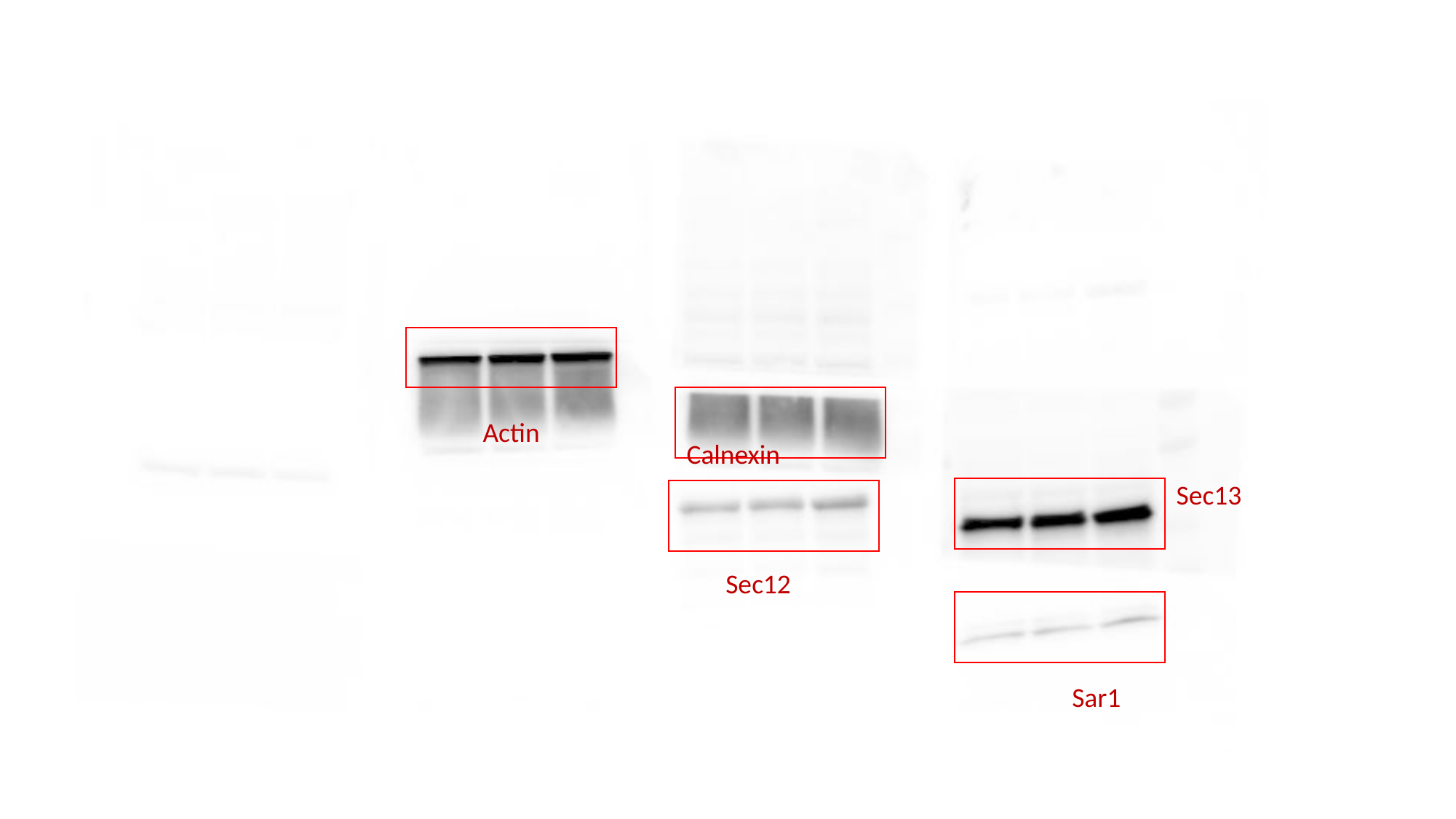

Actin
Calnexin
Sec13
Sec12
Sar1

Supplement: Supplementary file 9 — Source data Fig. 4 [file 44318_2024_208_MOESM9_ESM.zip › Figure4/Annotation-Figure4A.pptx]

## Slide 1
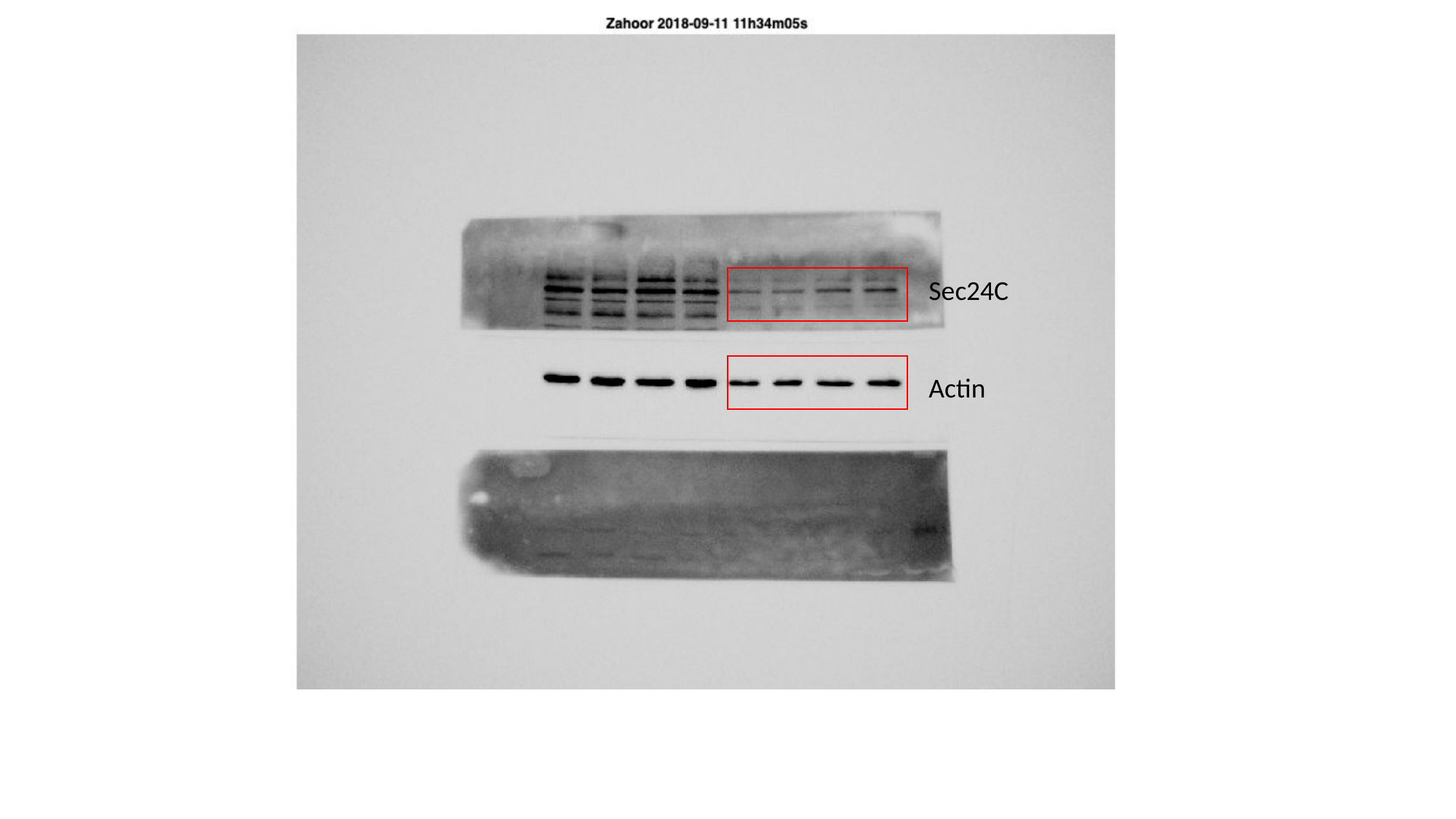

Sec24C
Actin

## Slide 2
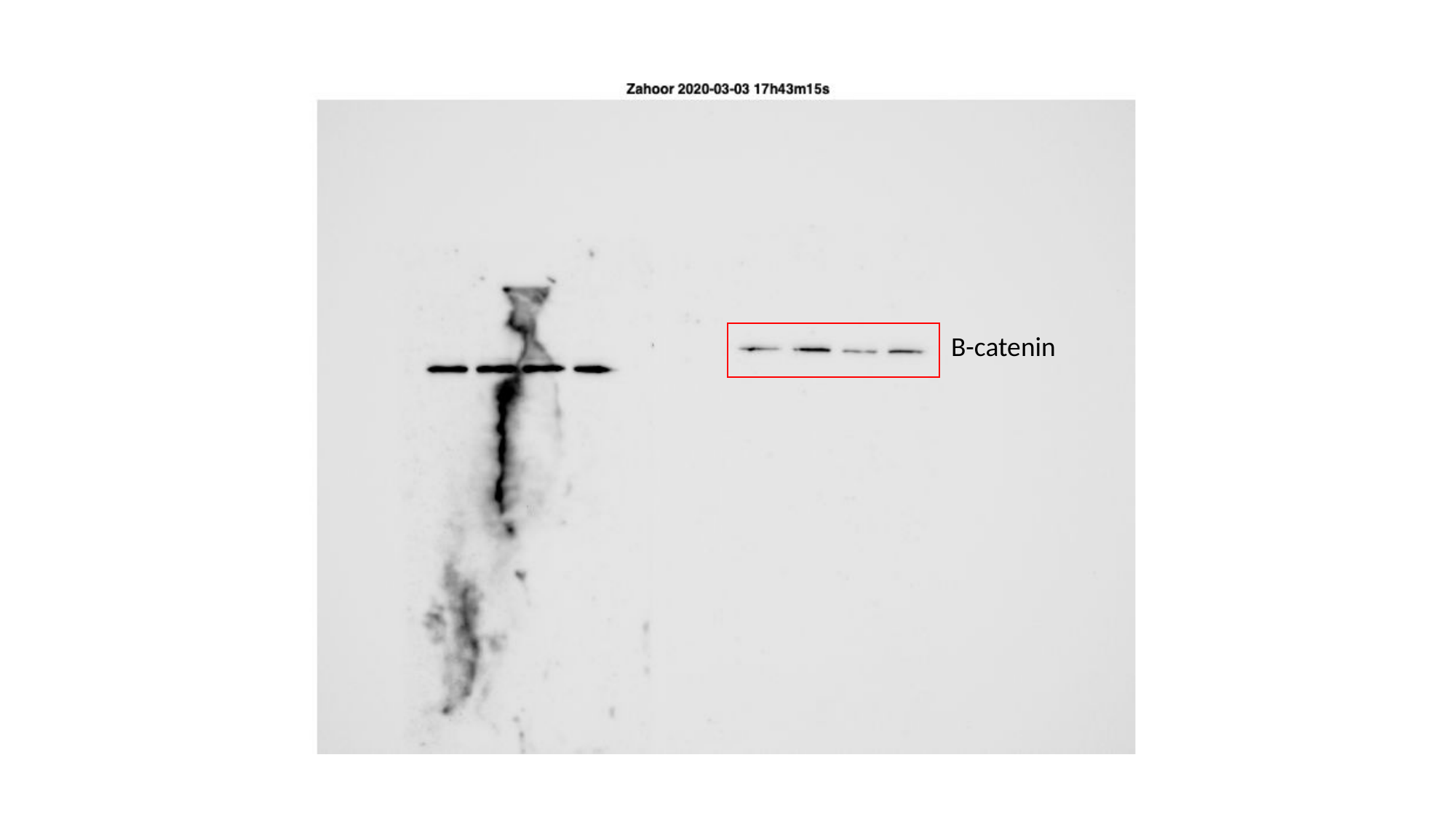

B-catenin

Supplement: Supplementary file 9 — Source data Fig. 4 [file 44318_2024_208_MOESM9_ESM.zip › Figure4/Annotation-Figure4C.pptx]

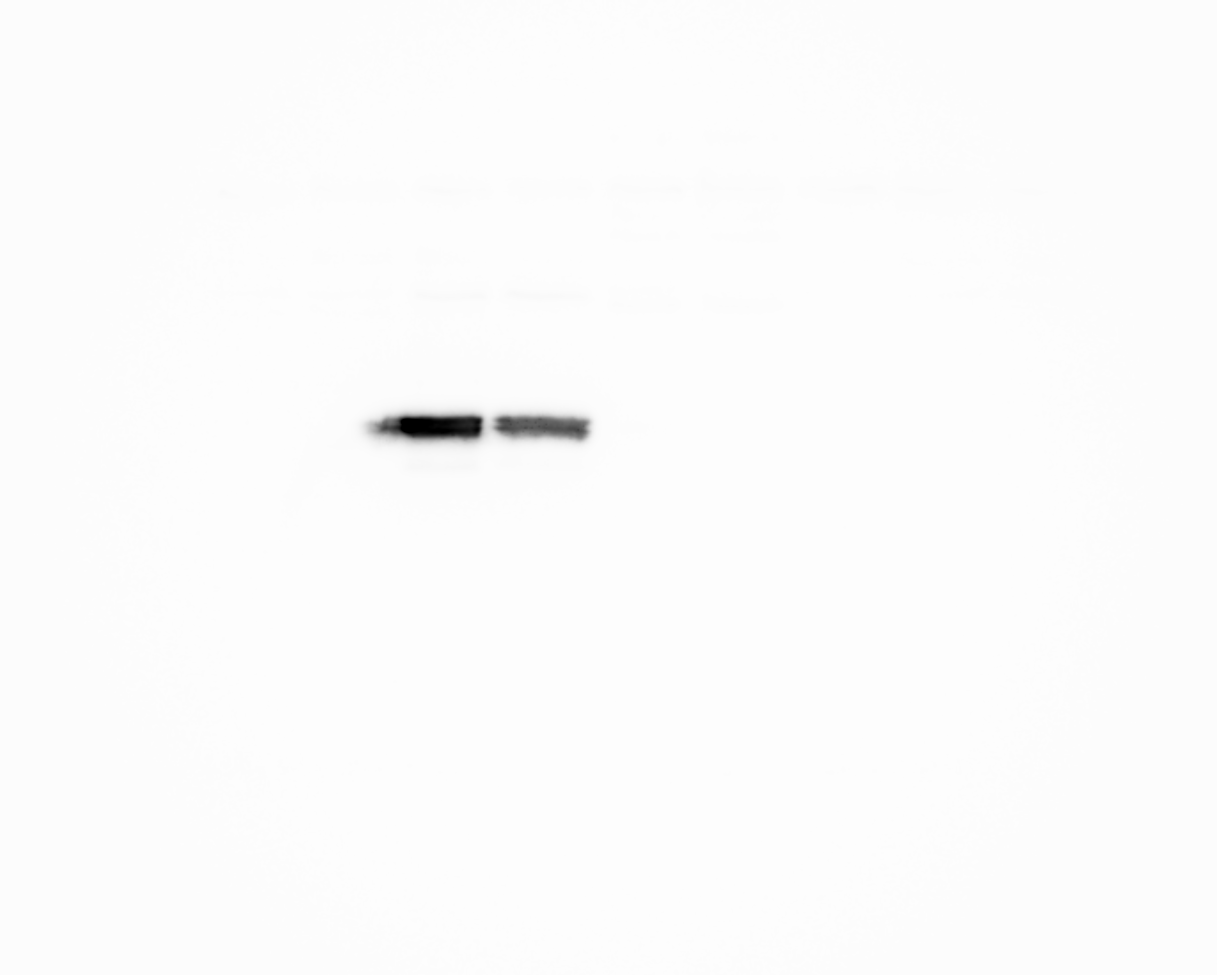

Supplement: Supplementary file 10 — Source data Fig. 5 [file 44318_2024_208_MOESM10_ESM.zip › Figure5/Figure5D_GFP.tif]

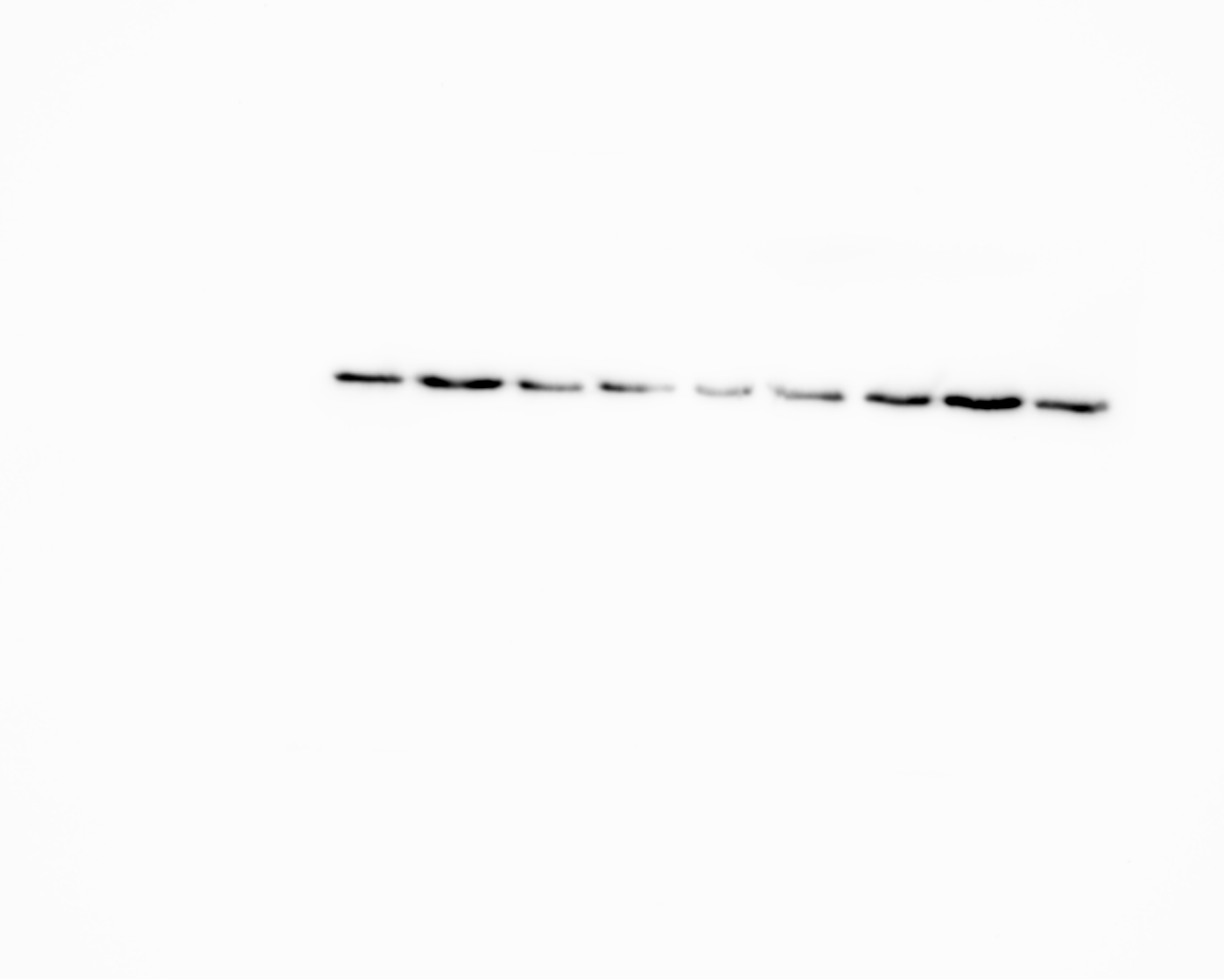

Supplement: Supplementary file 10 — Source data Fig. 5 [file 44318_2024_208_MOESM10_ESM.zip › Figure5/Figure5D_actin.tif]

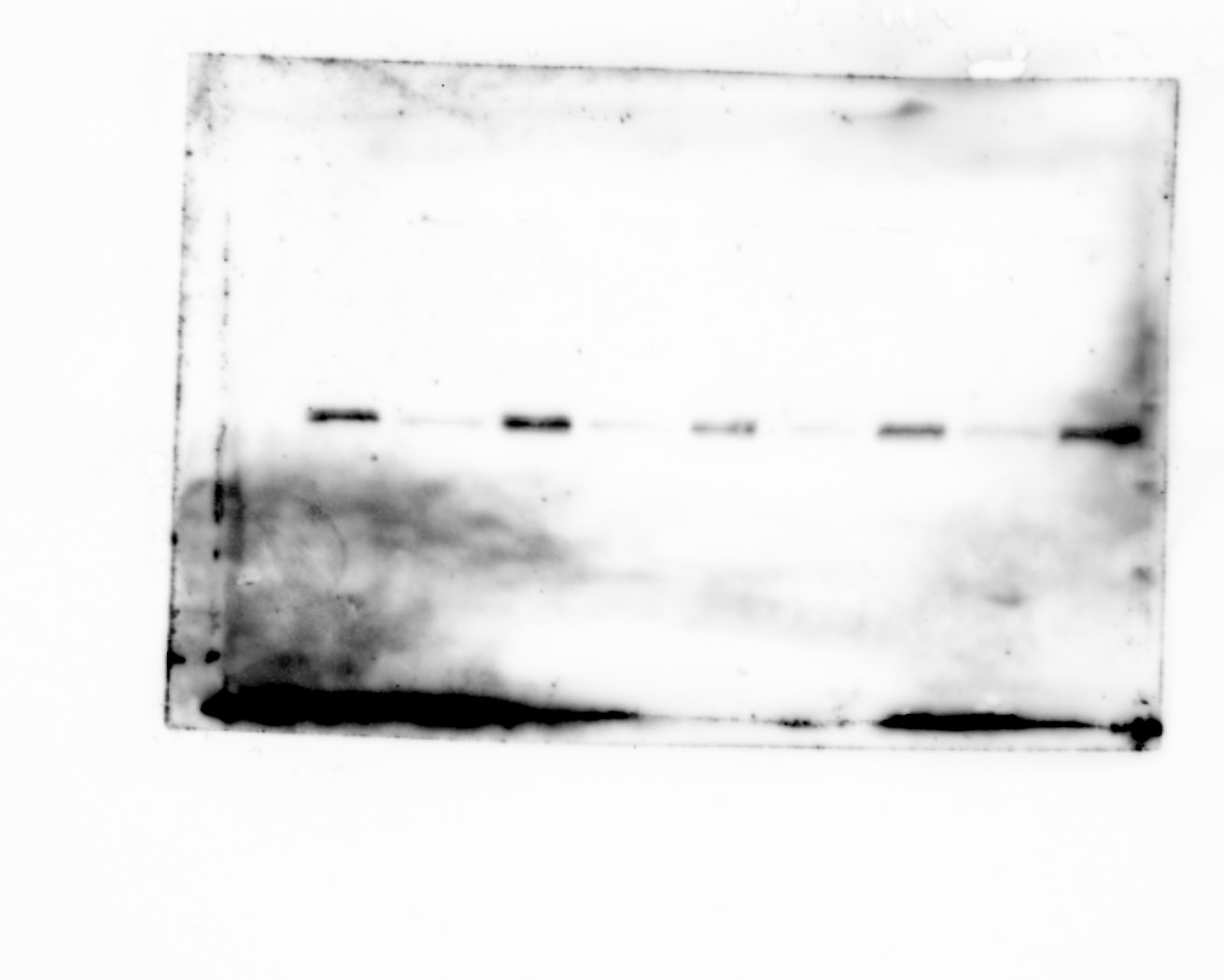

Supplement: Supplementary file 10 — Source data Fig. 5 [file 44318_2024_208_MOESM10_ESM.zip › Figure5/Figure5D_SNRPB.tif]

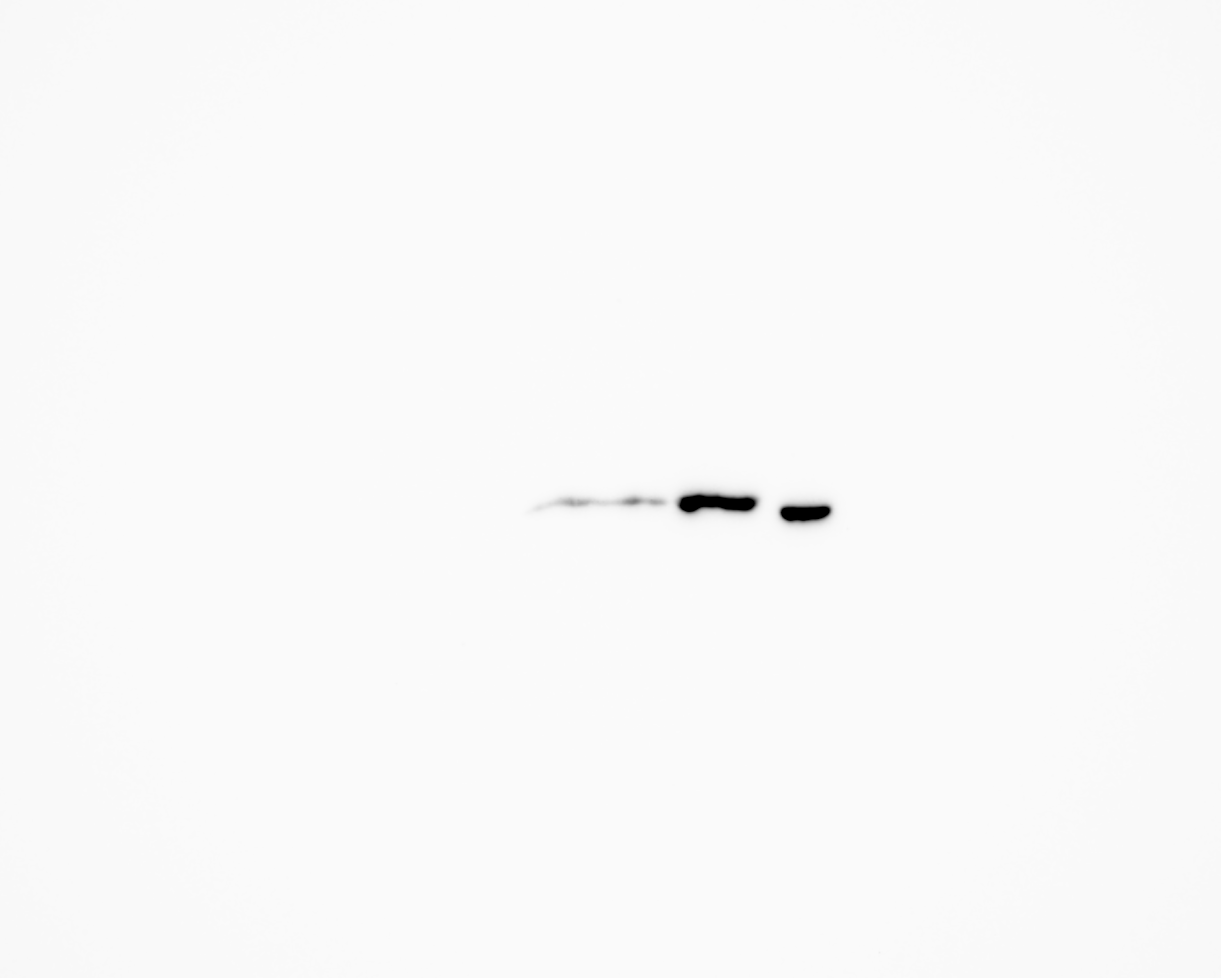

Supplement: Supplementary file 10 — Source data Fig. 5 [file 44318_2024_208_MOESM10_ESM.zip › Figure5/Figure5C_gapdh.tif]

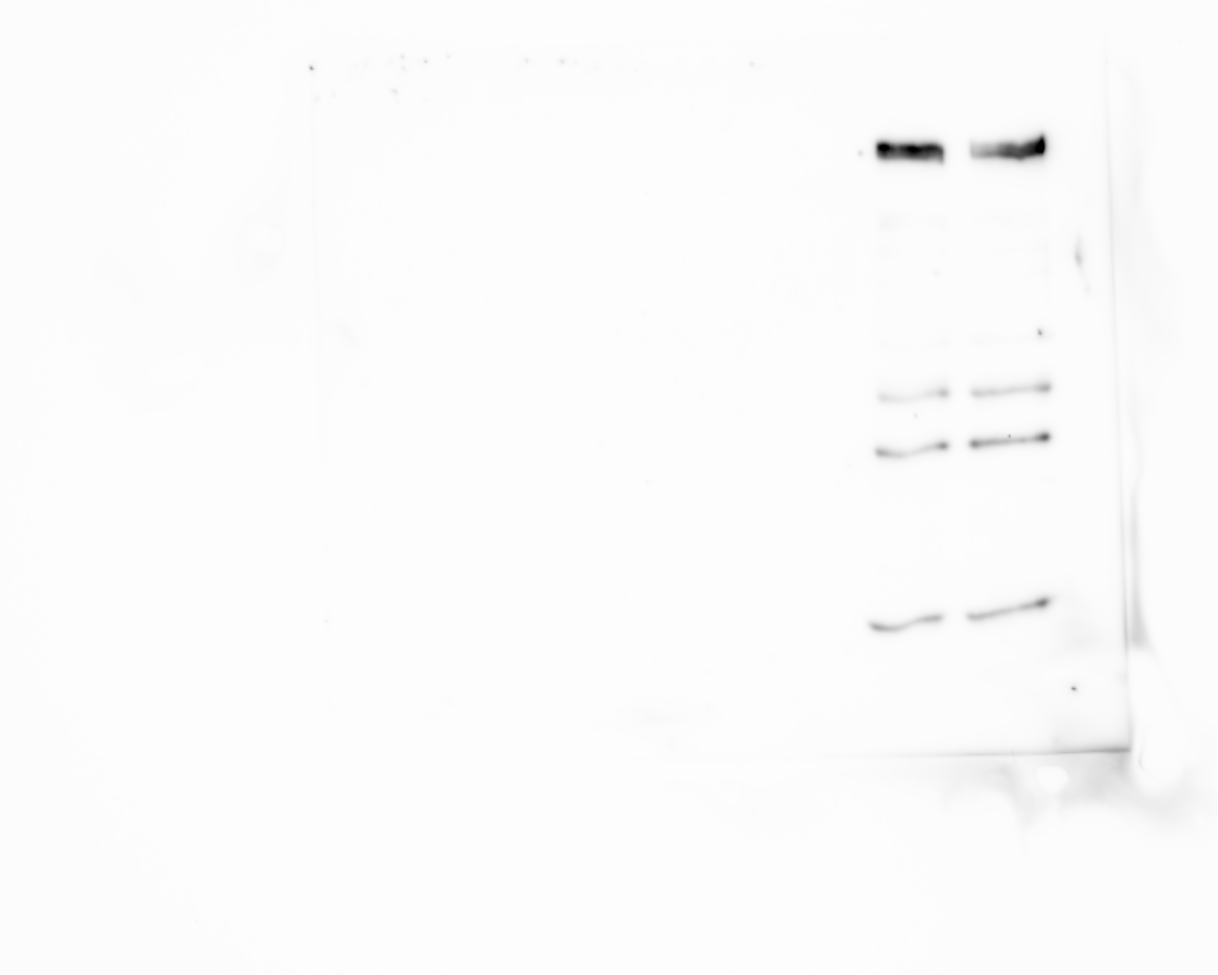

Supplement: Supplementary file 10 — Source data Fig. 5 [file 44318_2024_208_MOESM10_ESM.zip › Figure5/Figure 5D GFP-Sec16A.tif]

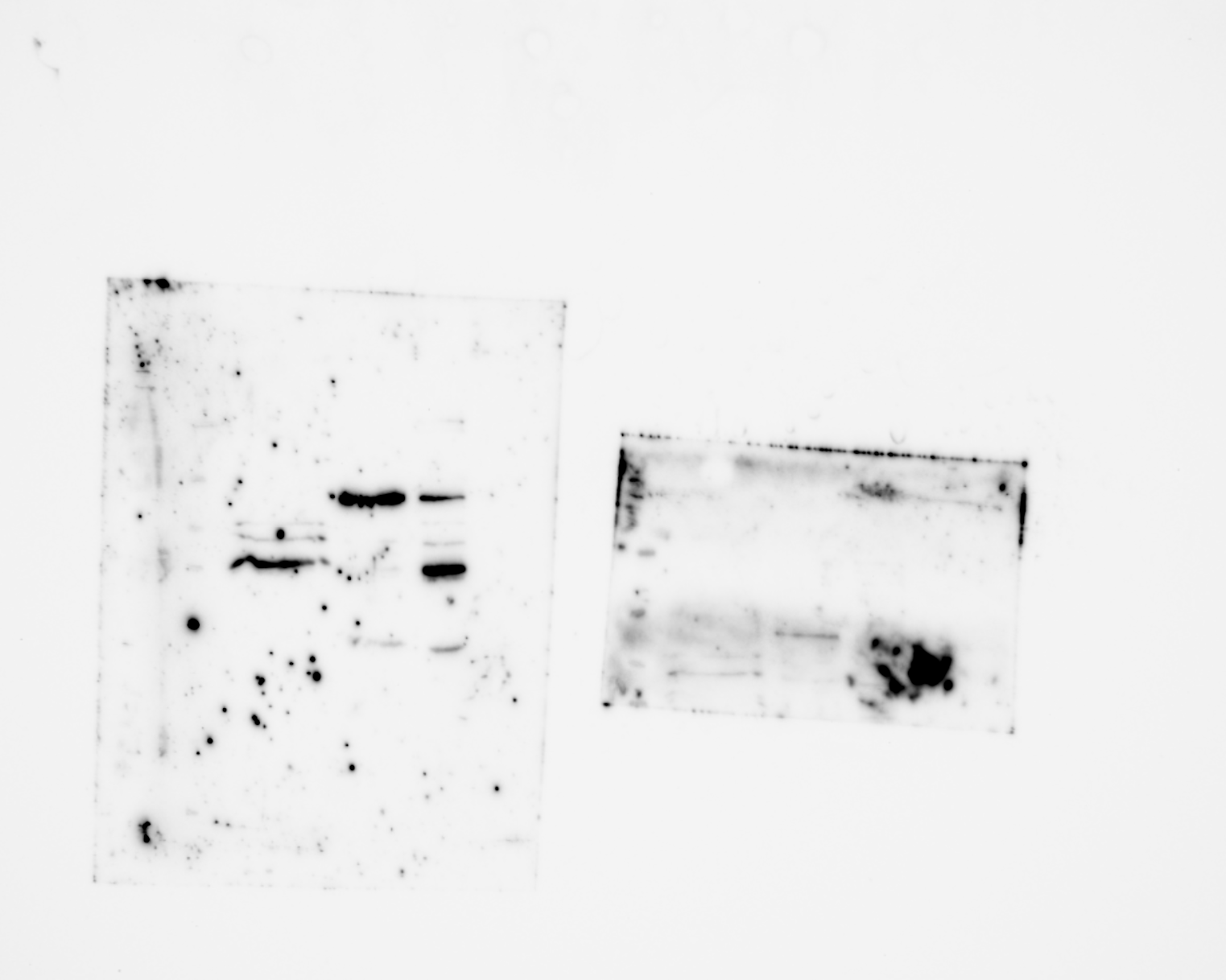

Supplement: Supplementary file 10 — Source data Fig. 5 [file 44318_2024_208_MOESM10_ESM.zip › Figure5/Figure5C_laminA.tif]

## Slide 1
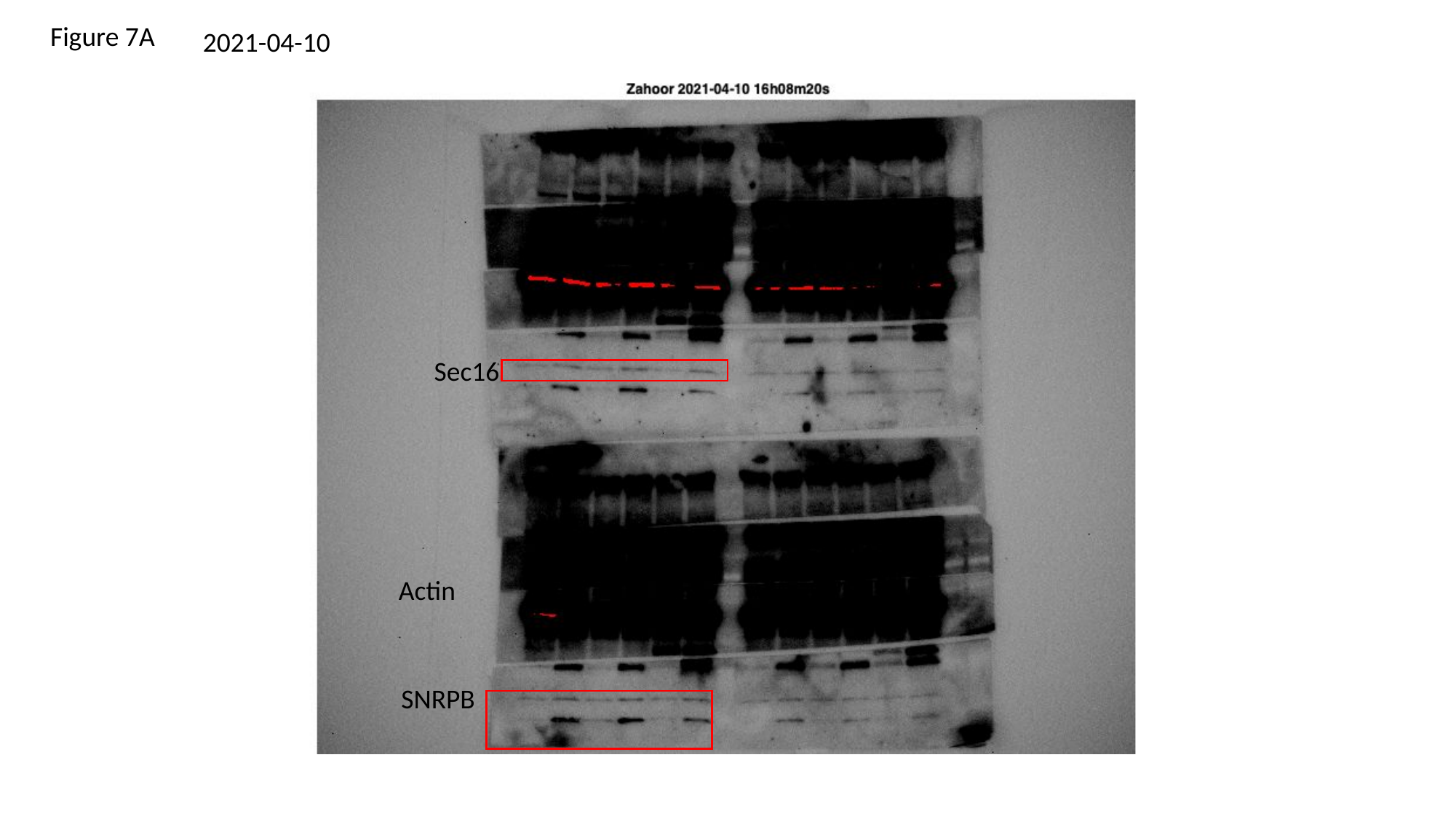

Figure 7A
2021-04-10
Sec16
Actin
SNRPB

## Slide 2
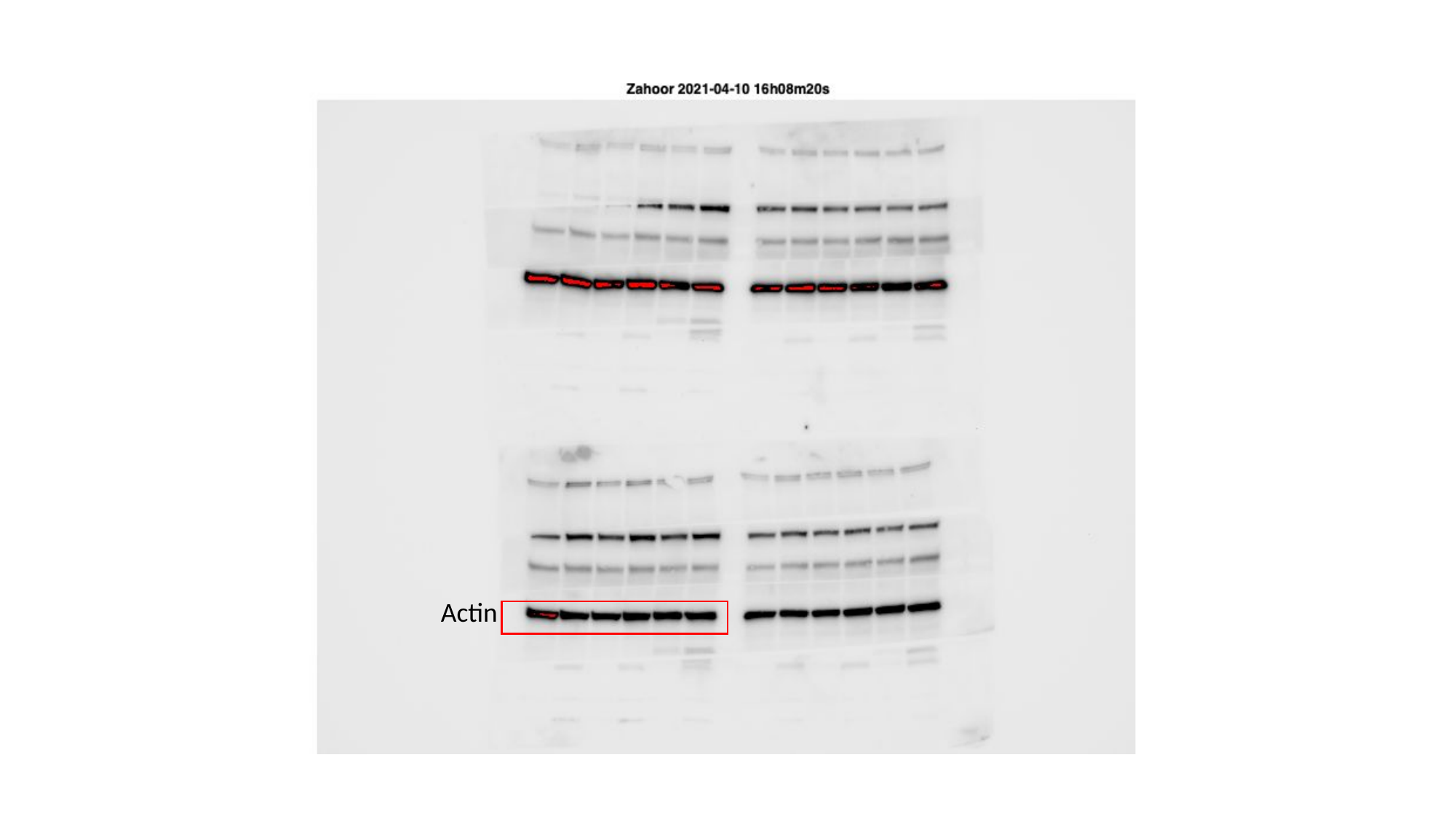

Actin

## Slide 3
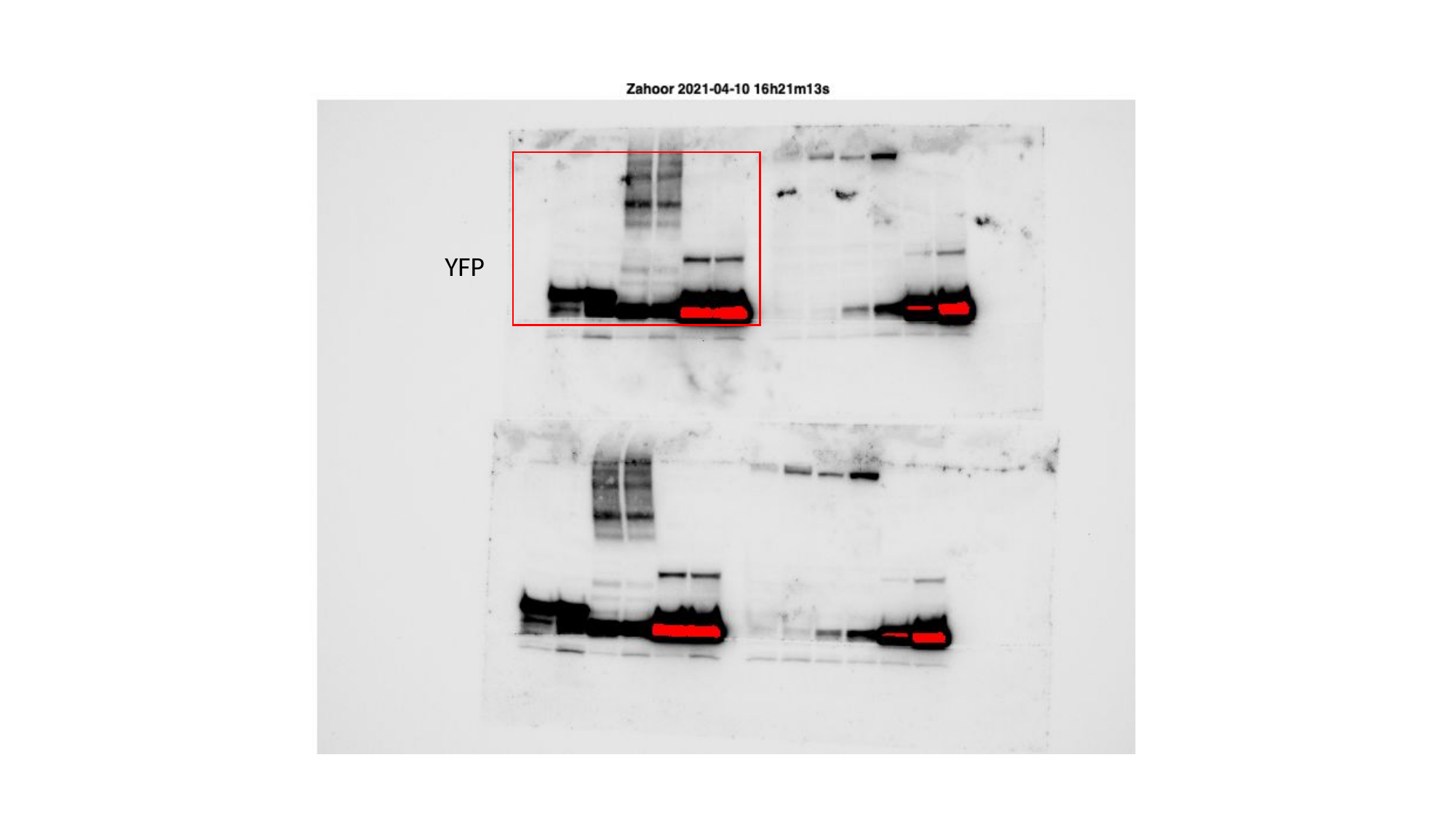

YFP

Supplement: Supplementary file 11 — Source data Fig. 7 [file 44318_2024_208_MOESM11_ESM.zip › Figure7/Annotation-Figure 7A.pptx]

## Slide 1
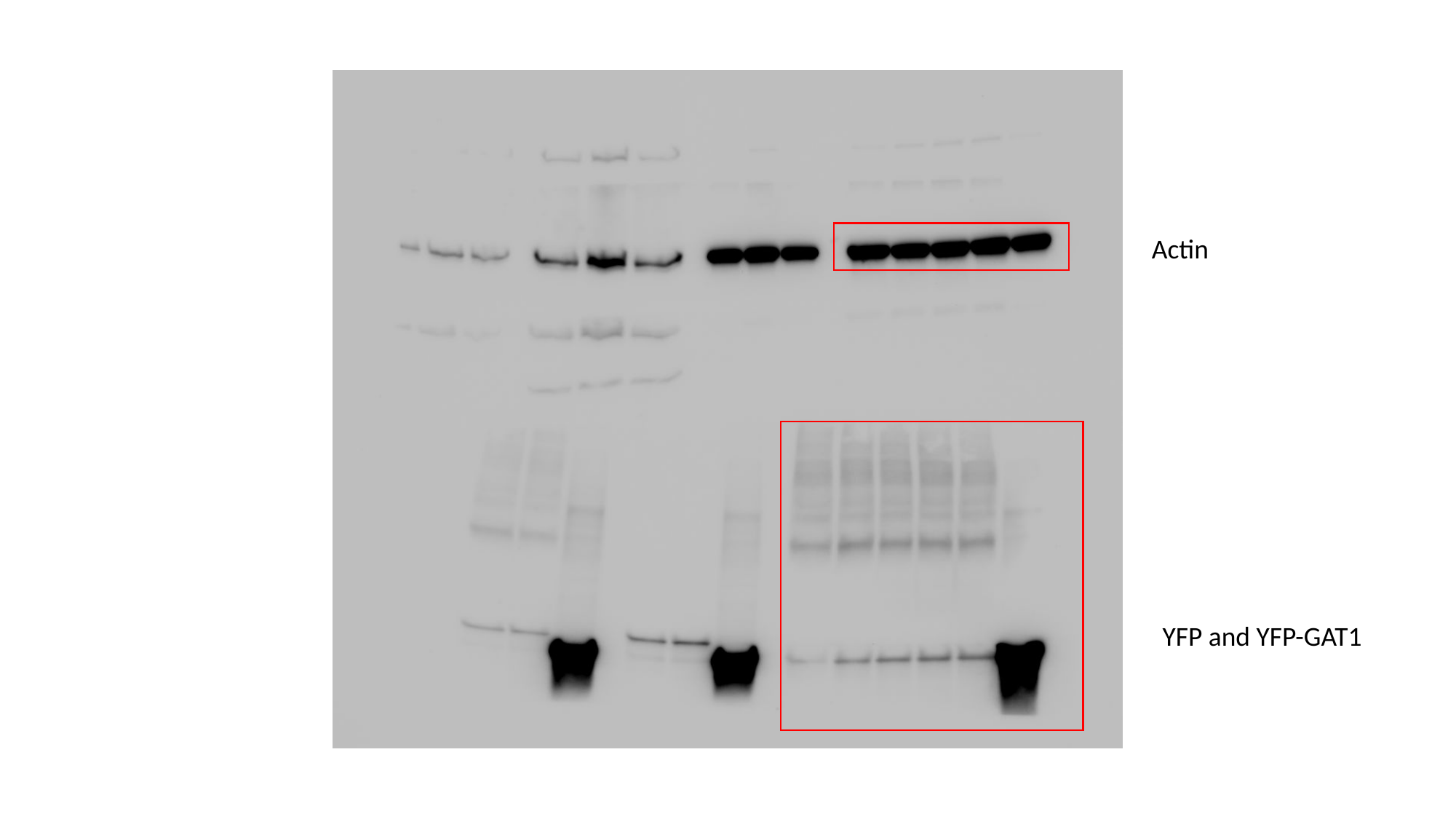

Actin
YFP and YFP-GAT1

## Slide 2
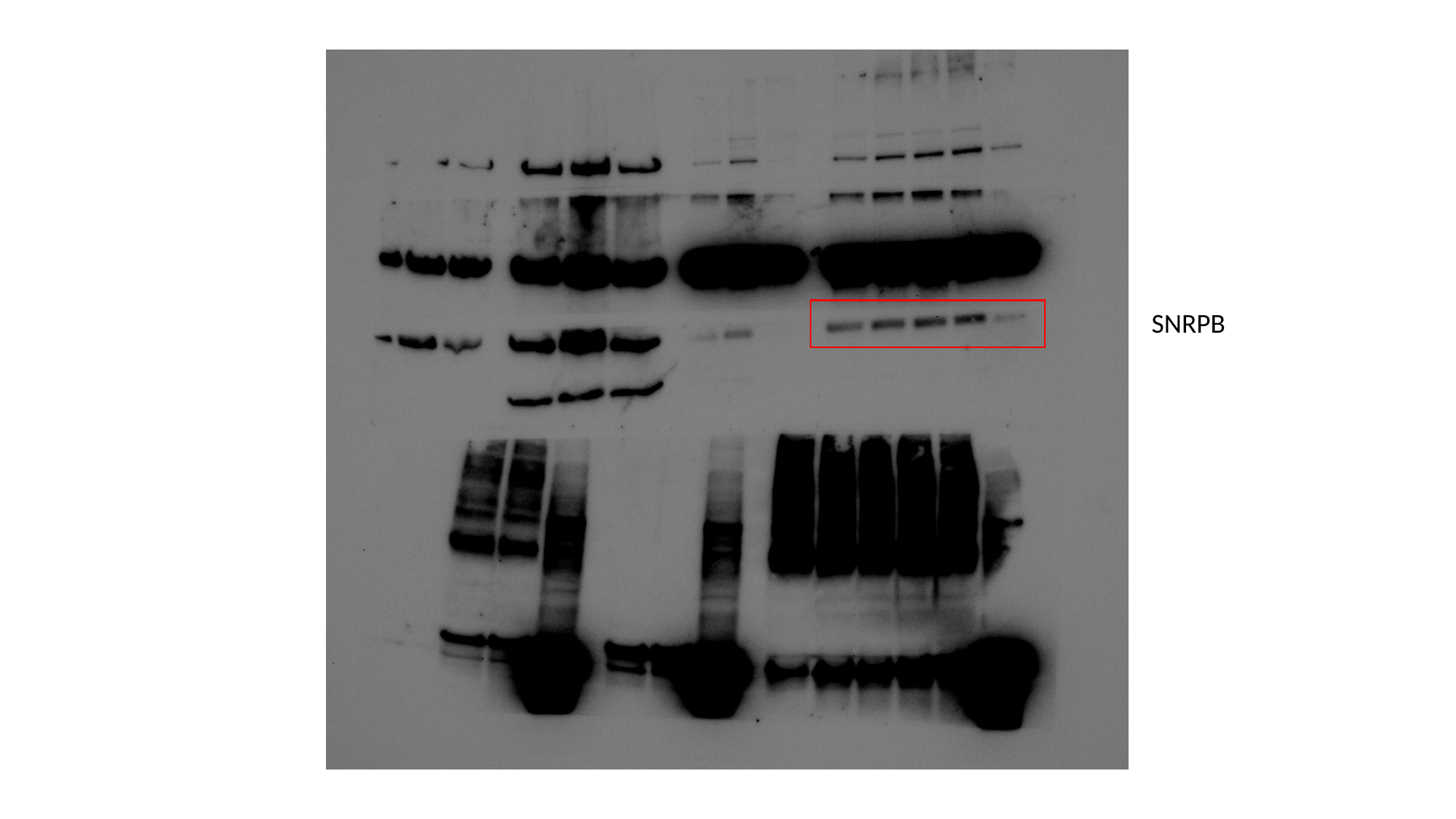

SNRPB

Supplement: Supplementary file 11 — Source data Fig. 7 [file 44318_2024_208_MOESM11_ESM.zip › Figure7/Annotation-Figure7D.pptx]

## Slide 1
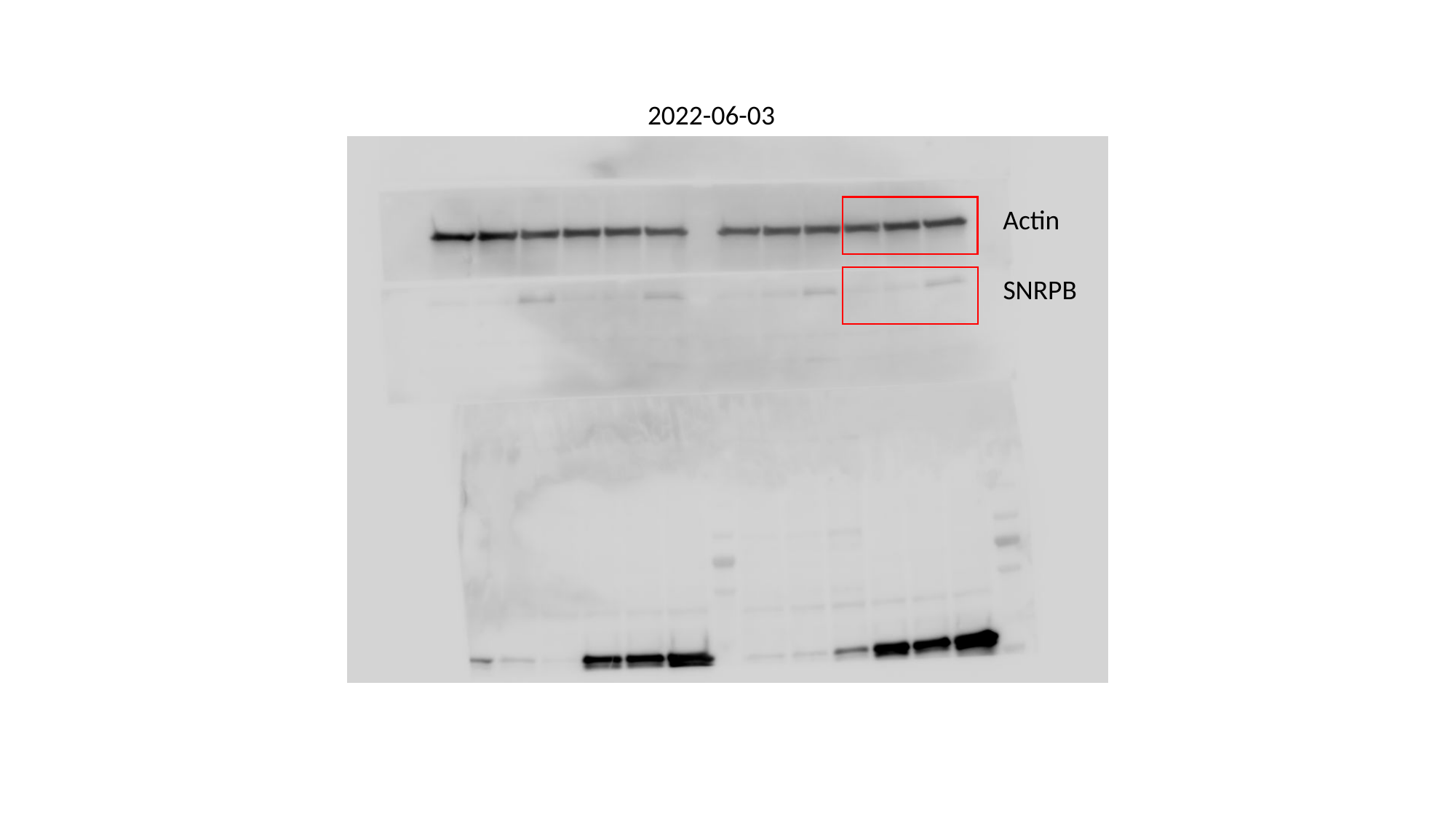

2022-06-03
Actin
SNRPB

## Slide 2
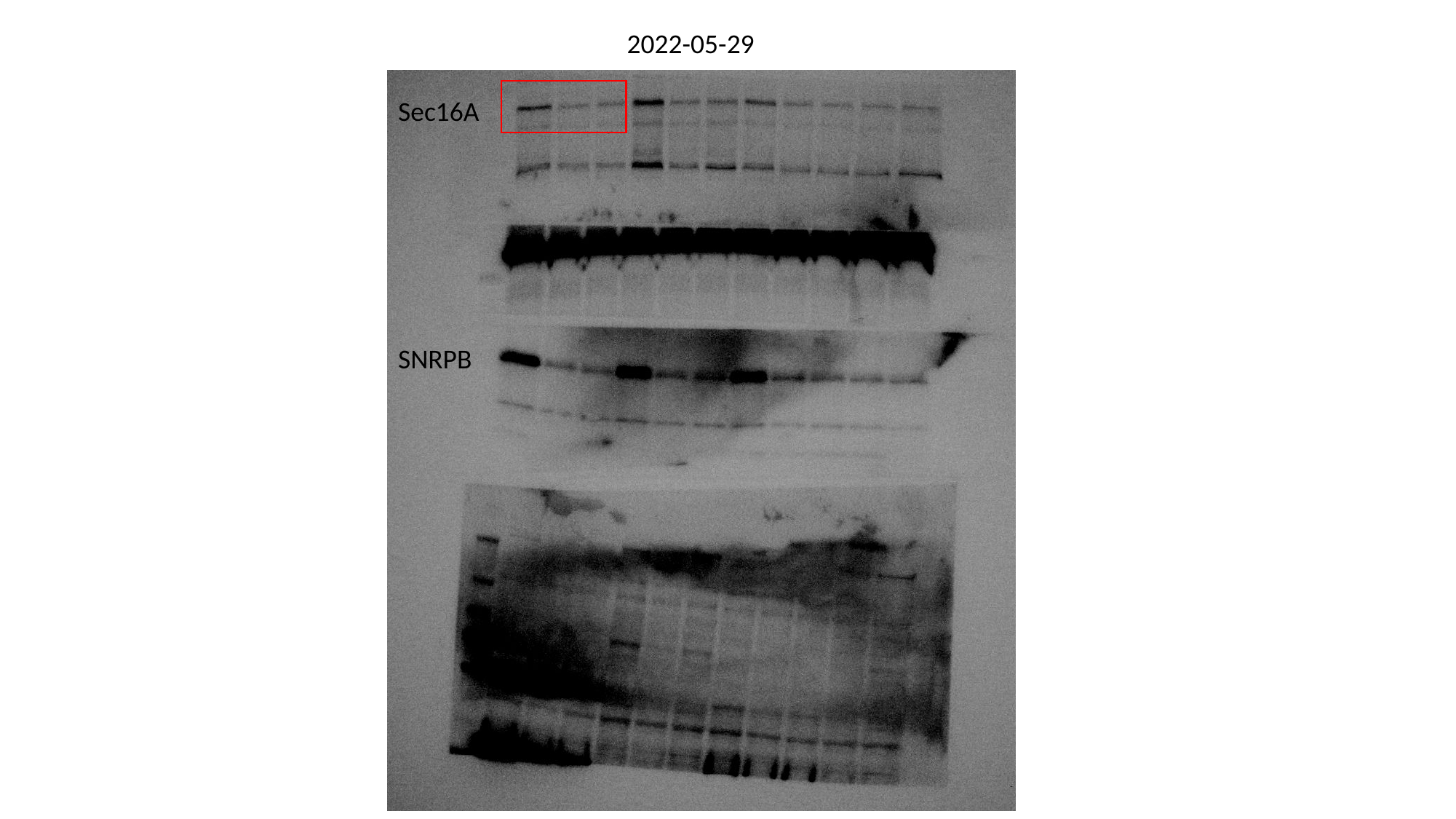

2022-05-29
Sec16A
SNRPB

Supplement: Supplementary file 12 — Source data Fig. 8 [file 44318_2024_208_MOESM12_ESM.zip › Figure8/Annotation-Figure8B.pptx]

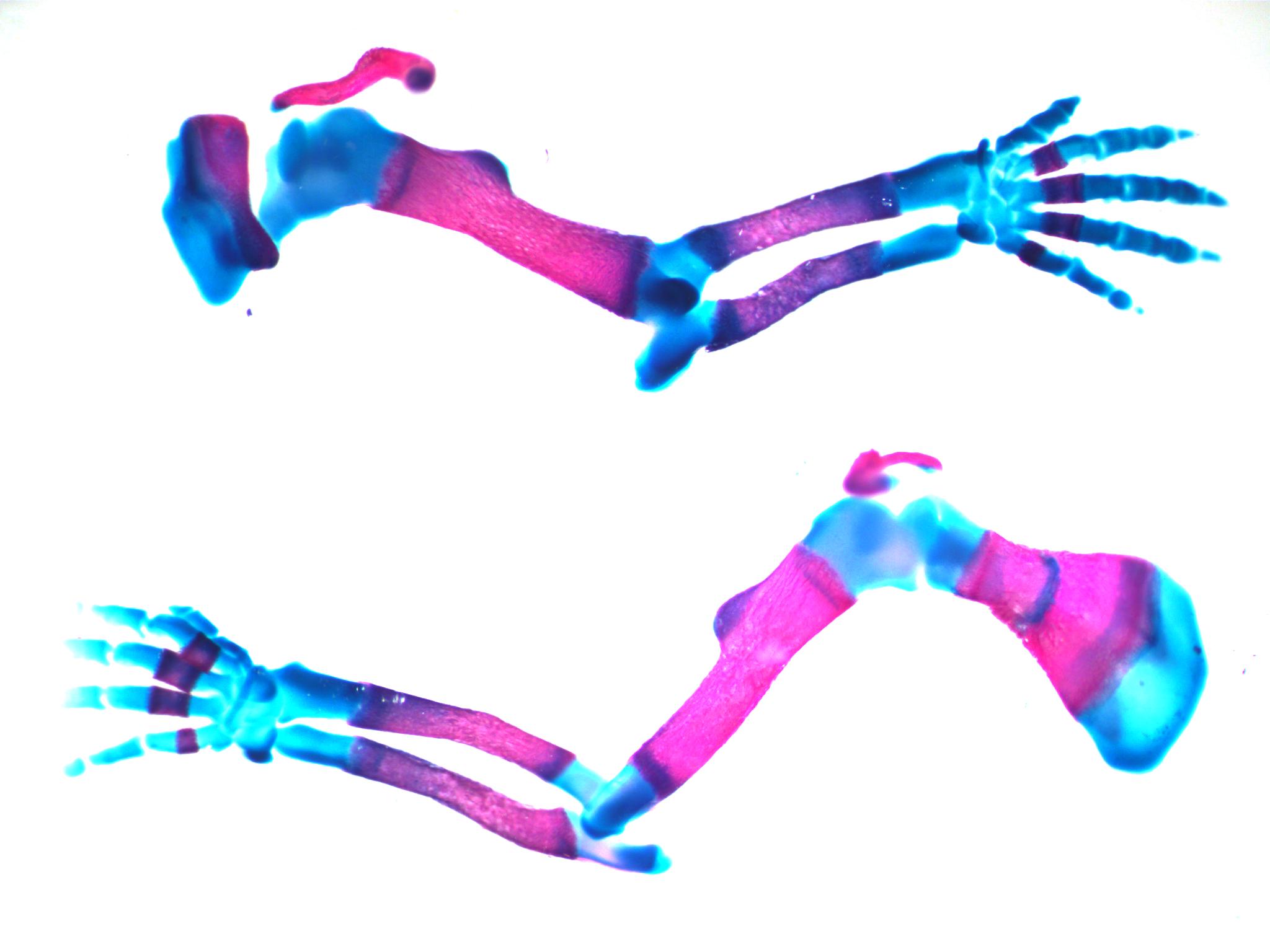

Supplement: Supplementary file 13 — Source data Fig. 9 [file 44318_2024_208_MOESM13_ESM.zip › Figure9/Figure 9C Mutant1.jpg]

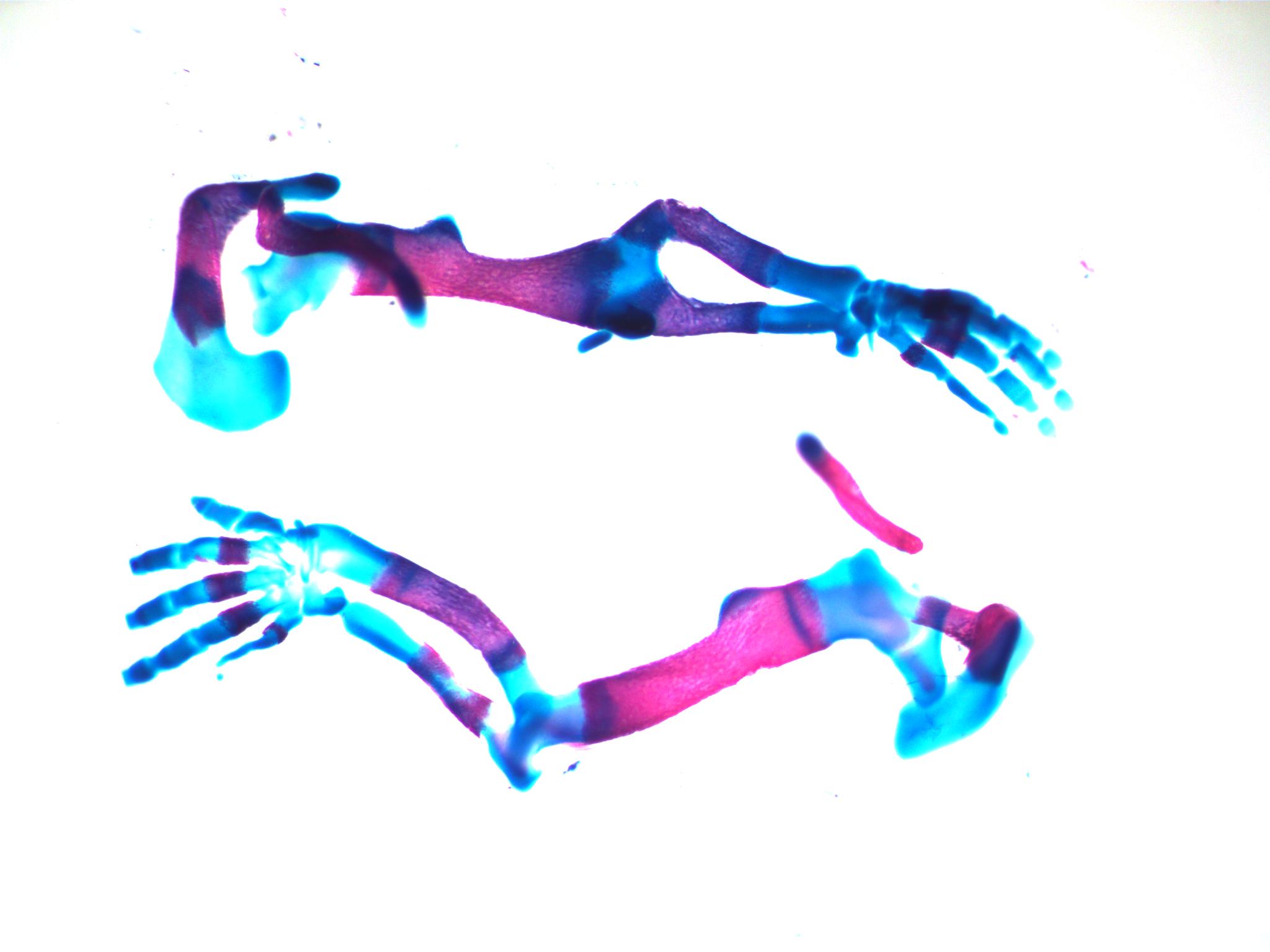

Supplement: Supplementary file 13 — Source data Fig. 9 [file 44318_2024_208_MOESM13_ESM.zip › Figure9/Figure 9C&D Mutant2.jpg]

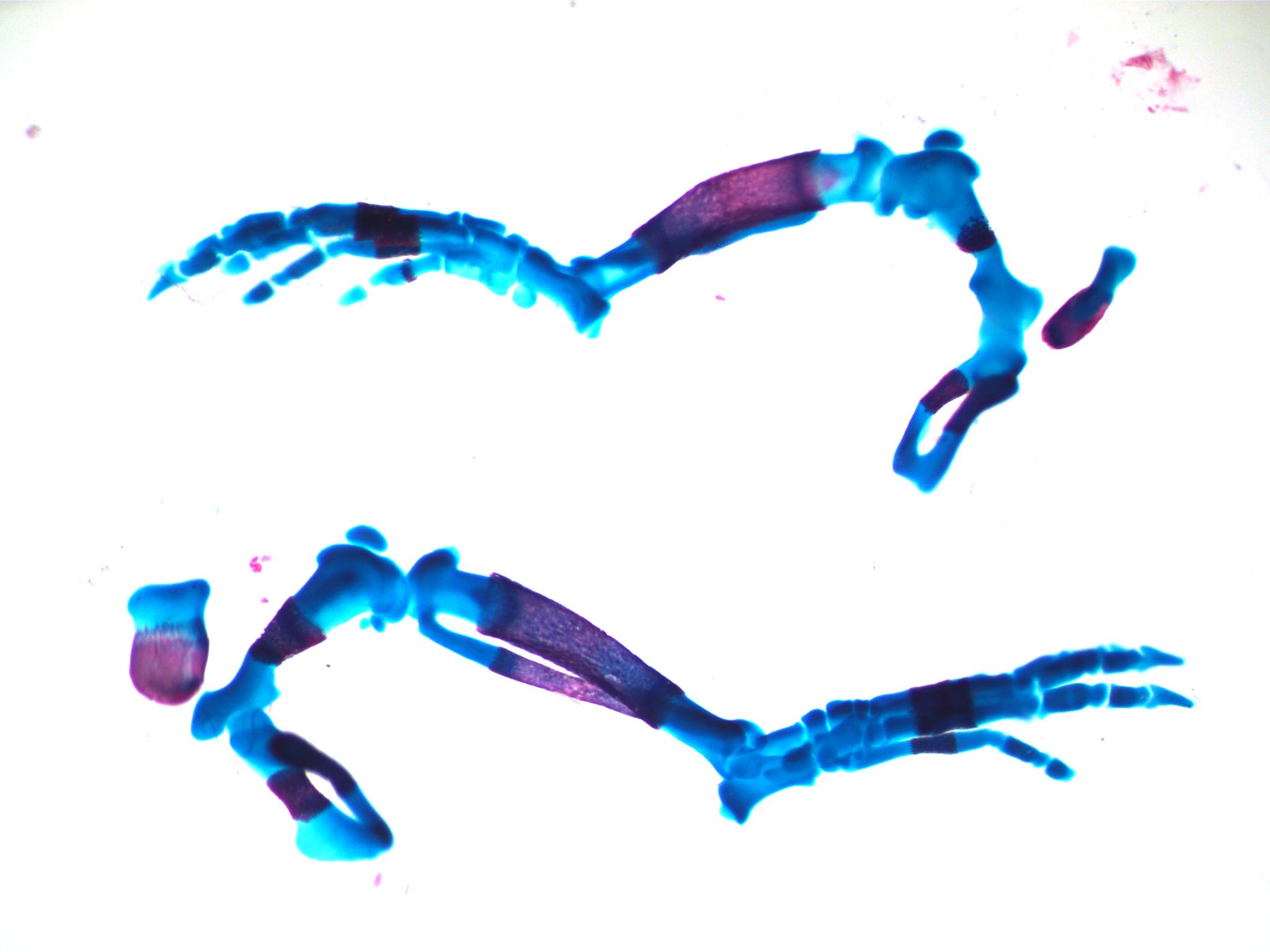

Supplement: Supplementary file 13 — Source data Fig. 9 [file 44318_2024_208_MOESM13_ESM.zip › Figure9/Figure 9F Mutant2.jpg]

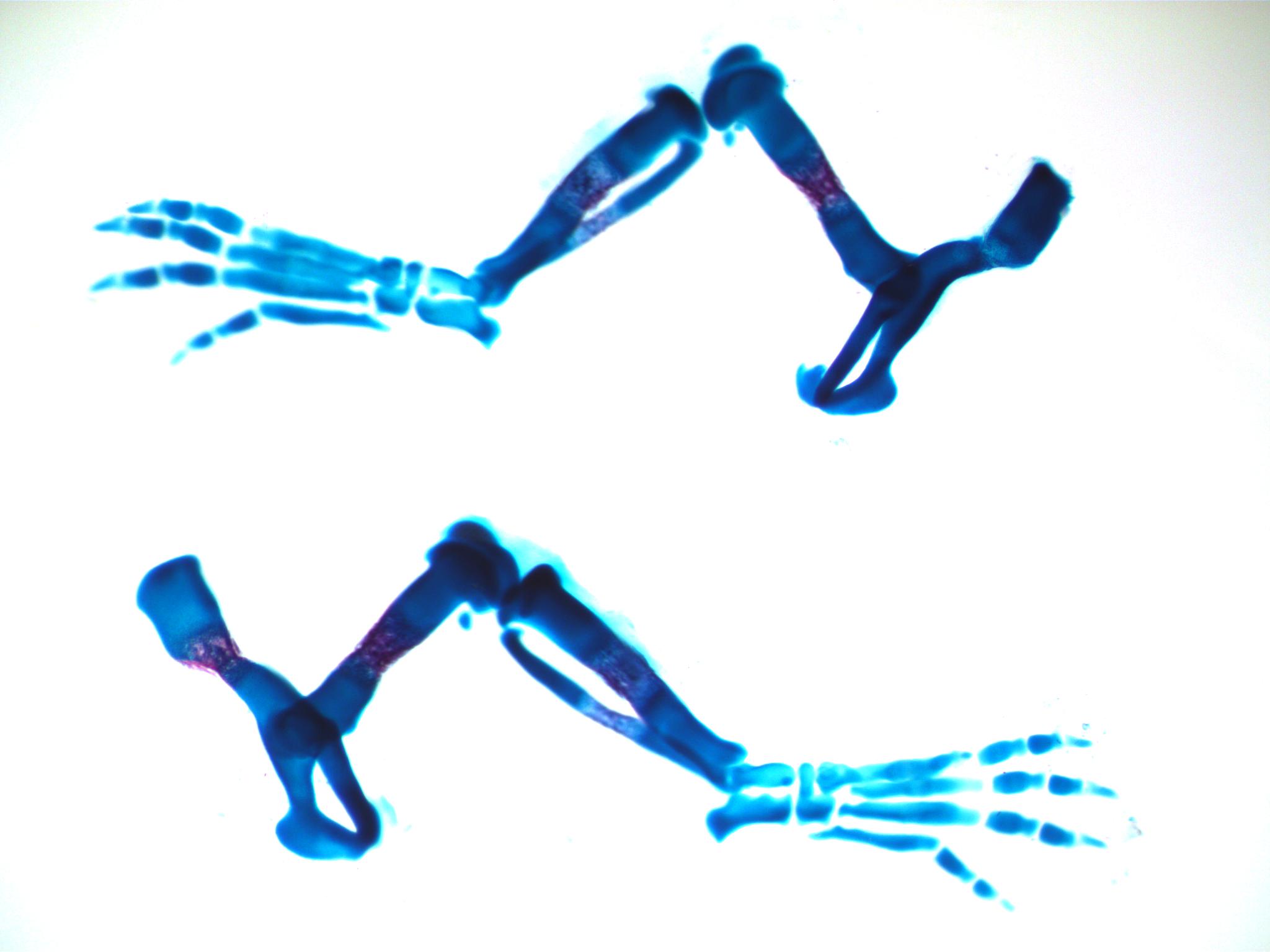

Supplement: Supplementary file 13 — Source data Fig. 9 [file 44318_2024_208_MOESM13_ESM.zip › Figure9/Figure 9F Mutant1.jpg]

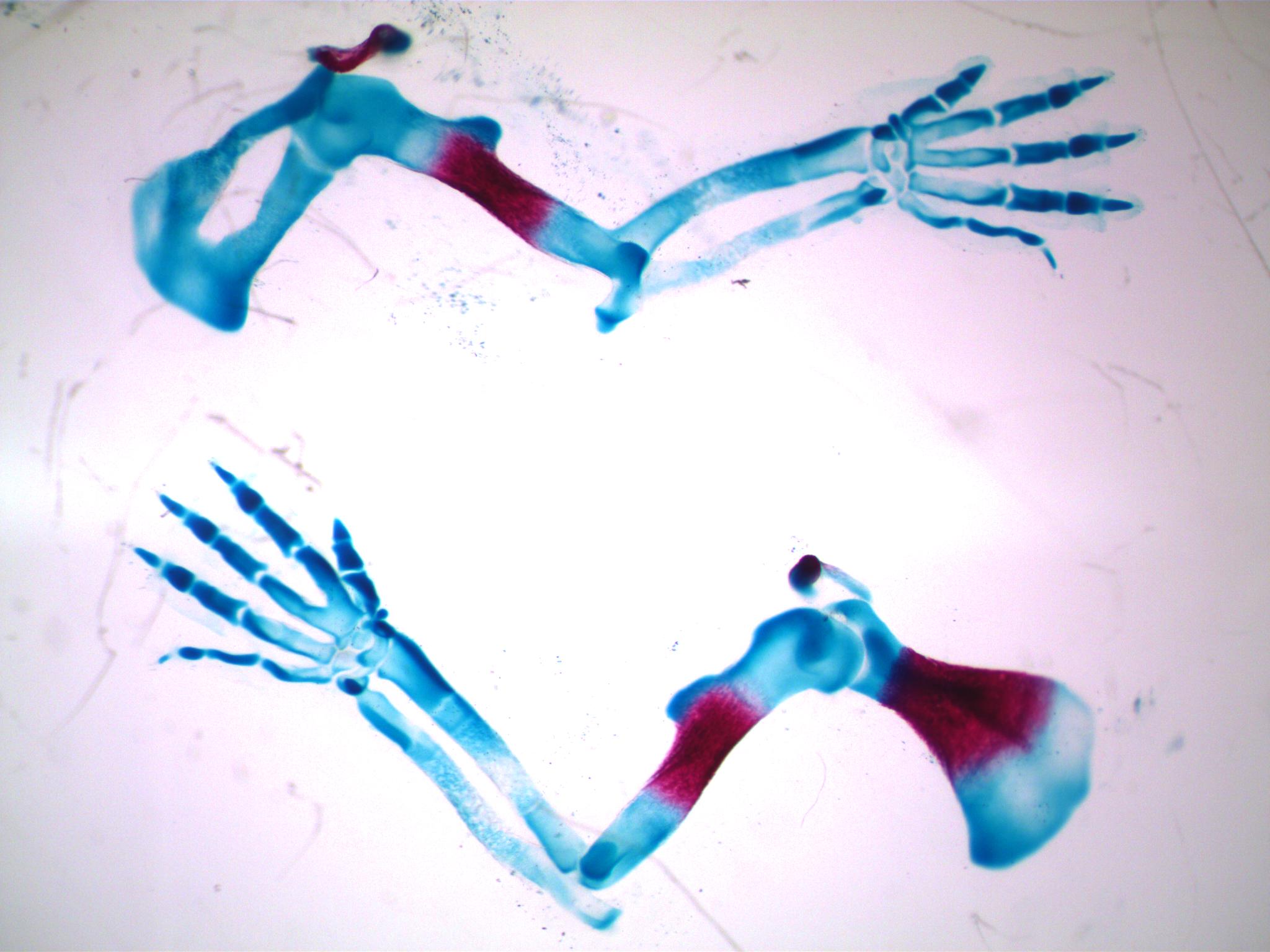

Supplement: Supplementary file 13 — Source data Fig. 9 [file 44318_2024_208_MOESM13_ESM.zip › Figure9/Figure 9E Mutant1.jpg]

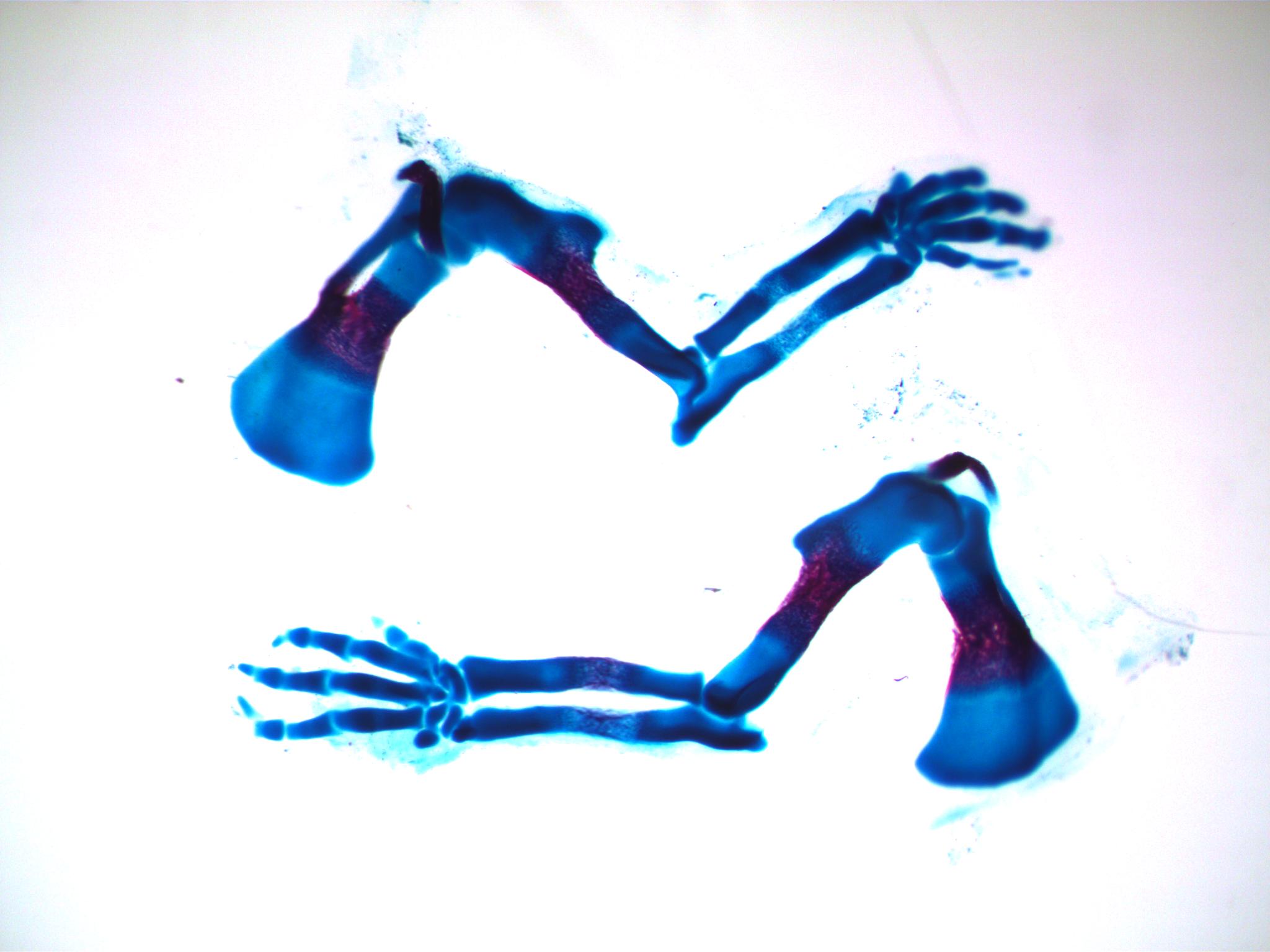

Supplement: Supplementary file 13 — Source data Fig. 9 [file 44318_2024_208_MOESM13_ESM.zip › Figure9/Figure 9E Mutant2.jpg]

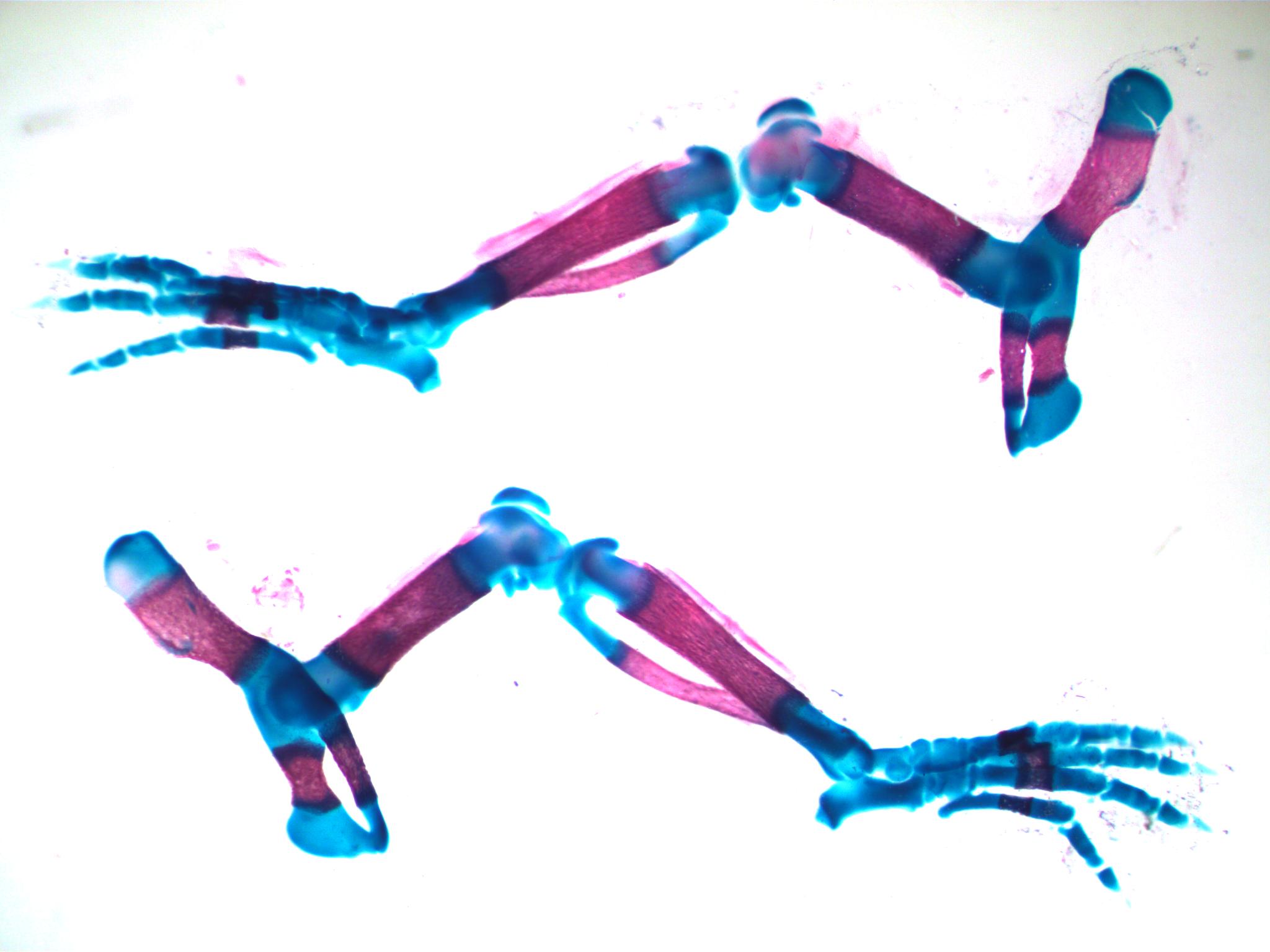

Supplement: Supplementary file 13 — Source data Fig. 9 [file 44318_2024_208_MOESM13_ESM.zip › Figure9/Figure 9F Control.jpg]

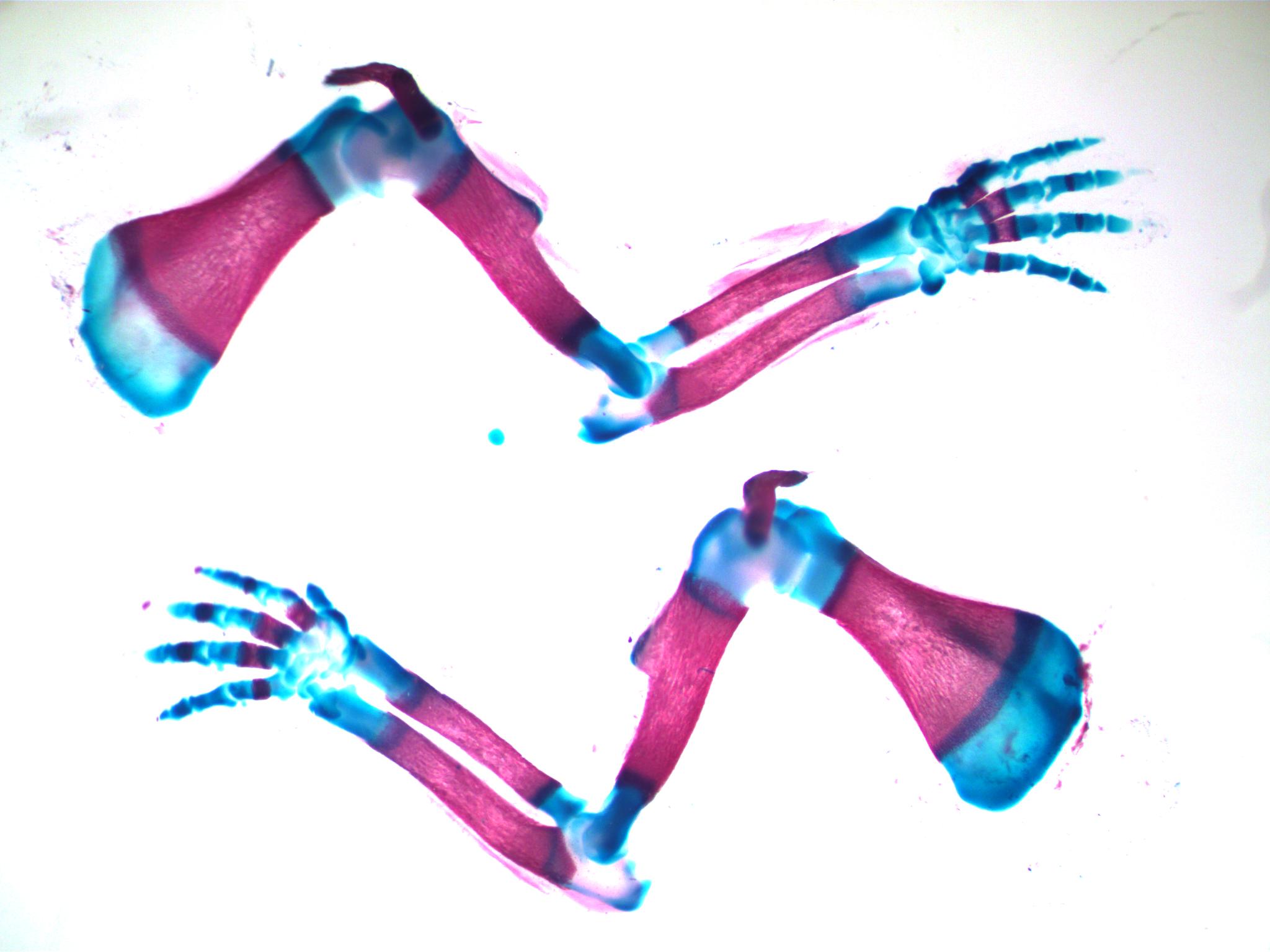

Supplement: Supplementary file 13 — Source data Fig. 9 [file 44318_2024_208_MOESM13_ESM.zip › Figure9/Figure 9C&D Control.jpg]

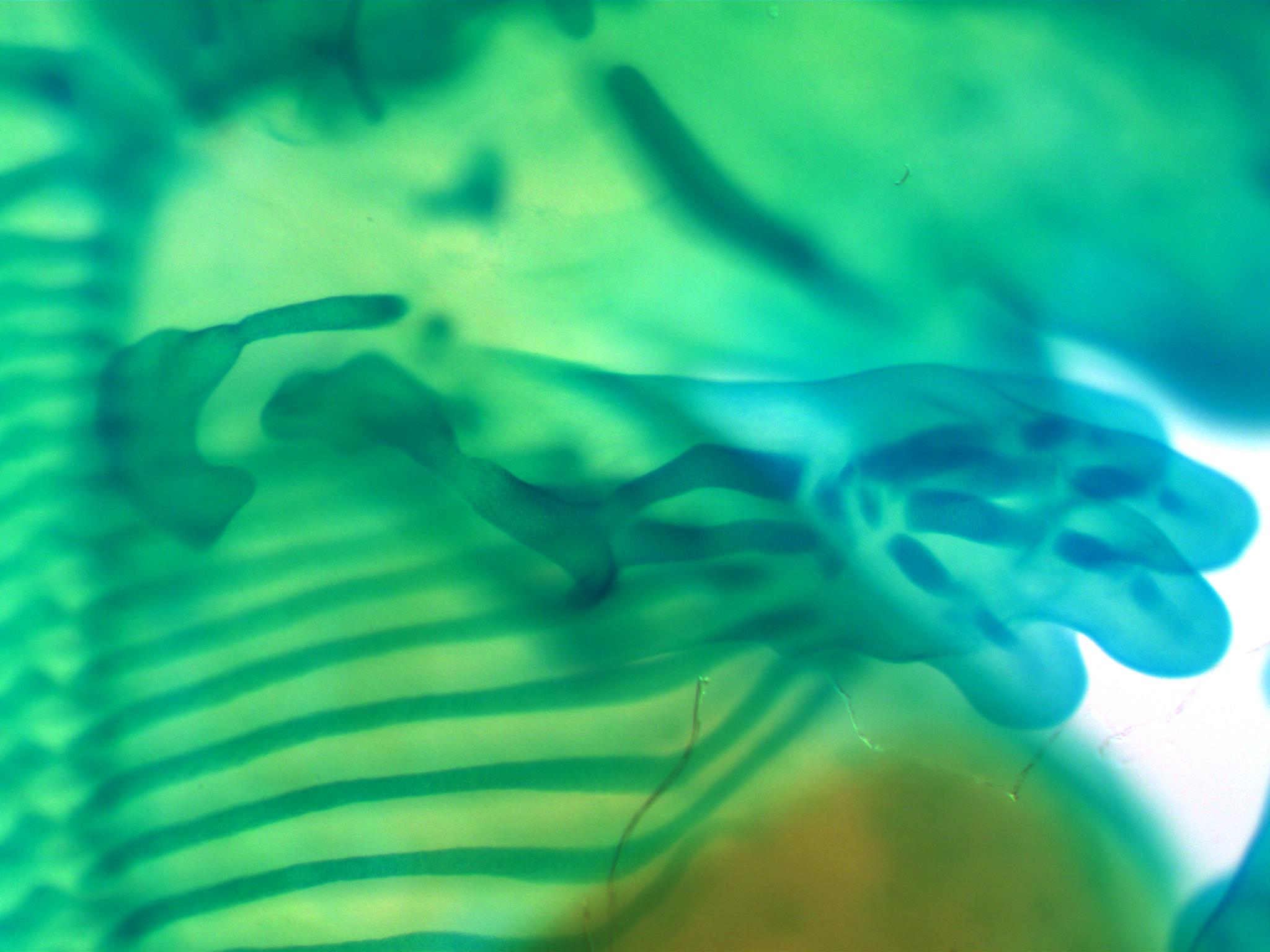

Supplement: Supplementary file 13 — Source data Fig. 9 [file 44318_2024_208_MOESM13_ESM.zip › Figure9/Figure 9A Mutant.jpg]

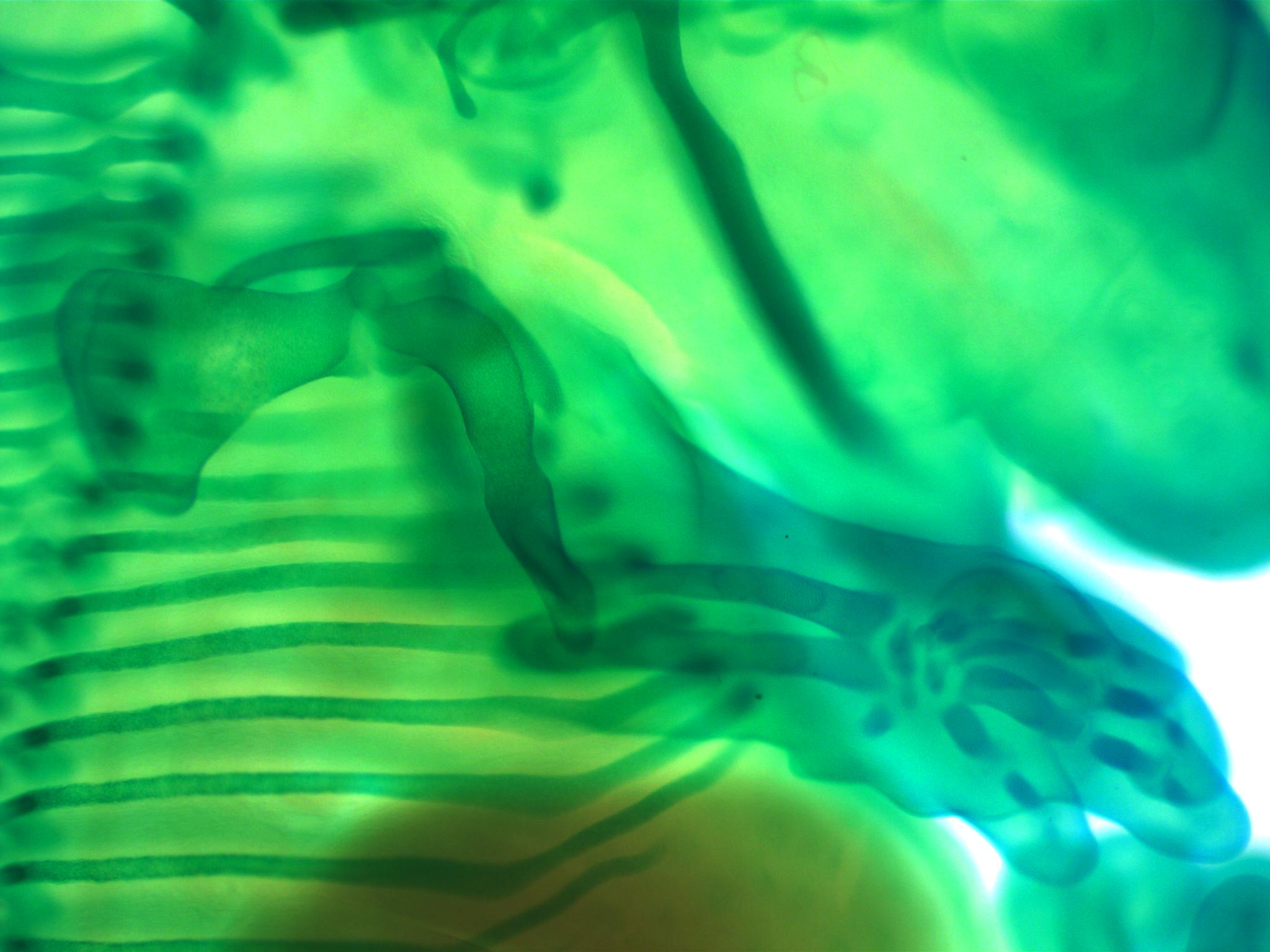

Supplement: Supplementary file 13 — Source data Fig. 9 [file 44318_2024_208_MOESM13_ESM.zip › Figure9/Figure 9A Control.jpg]

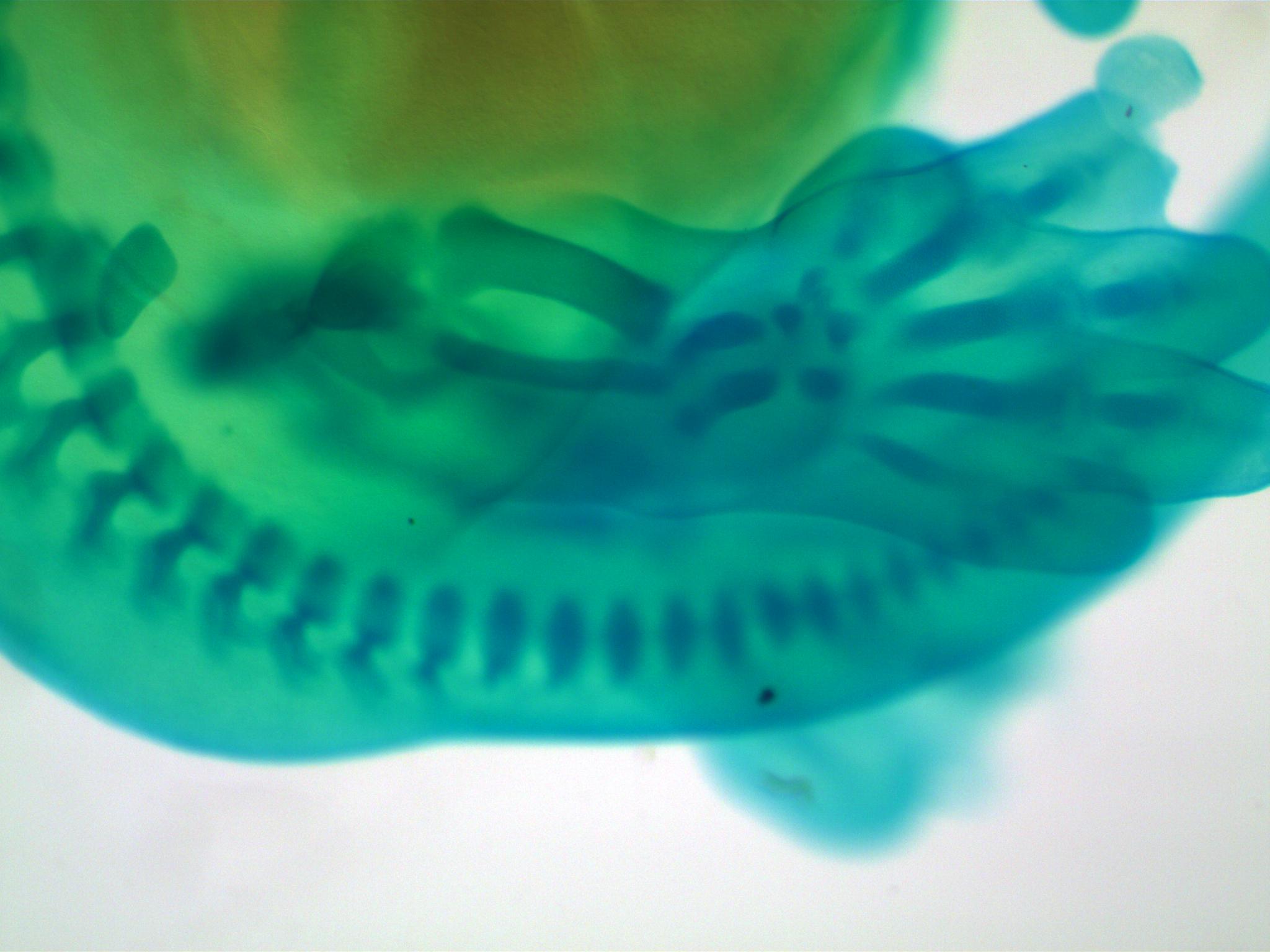

Supplement: Supplementary file 13 — Source data Fig. 9 [file 44318_2024_208_MOESM13_ESM.zip › Figure9/Figure 9B Mutant.jpg]

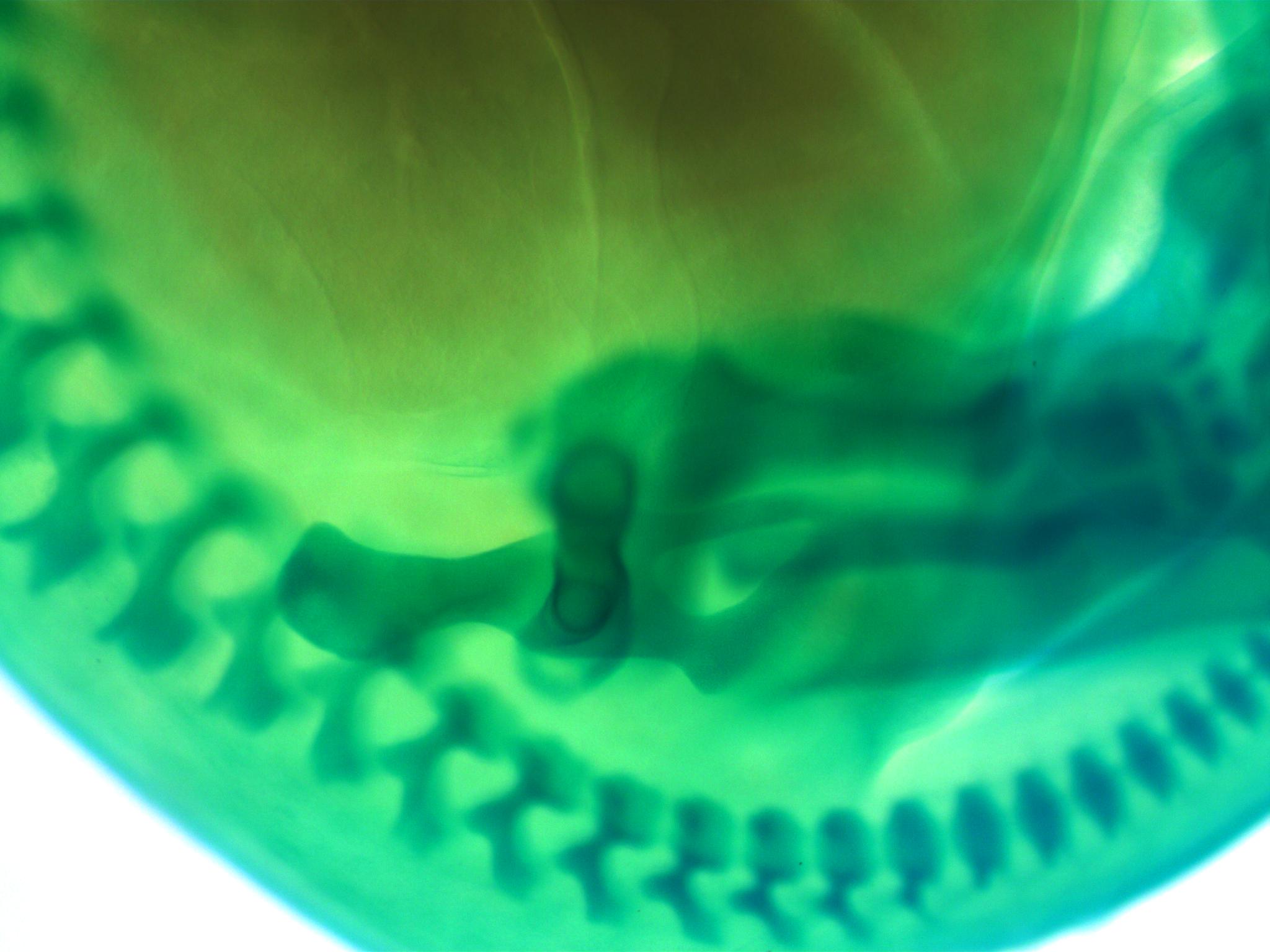

Supplement: Supplementary file 13 — Source data Fig. 9 [file 44318_2024_208_MOESM13_ESM.zip › Figure9/Figure 9B Control.jpg]

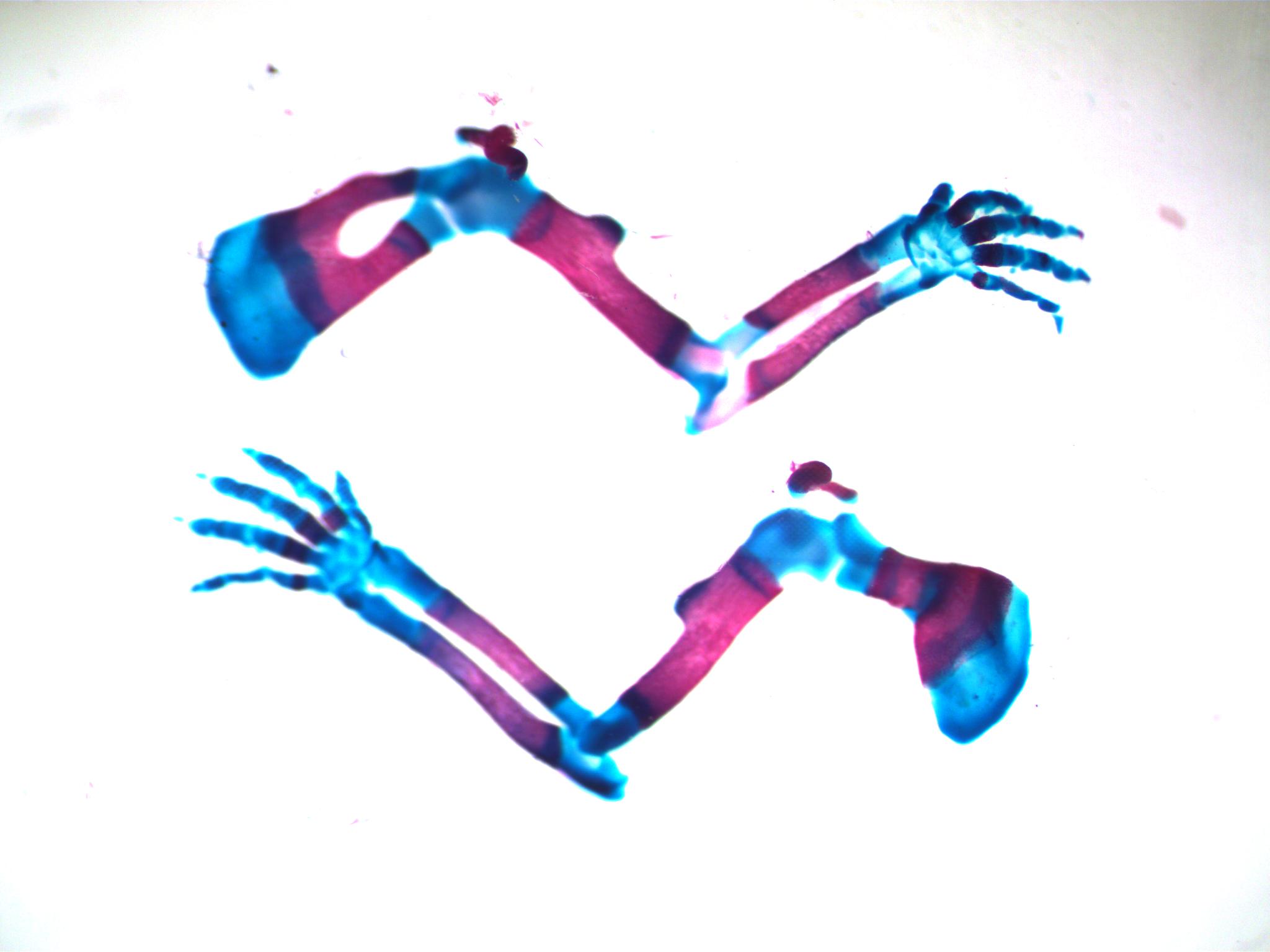

Supplement: Supplementary file 13 — Source data Fig. 9 [file 44318_2024_208_MOESM13_ESM.zip › Figure9/Figure 9D Mutant1.jpg]
